# Resolving complex structural genomic rearrangements using a randomized approach

**DOI:** 10.1186/s13059-016-0993-1

**Published:** 2016-06-10

**Authors:** Xuefang Zhao, Sarah B. Emery, Bridget Myers, Jeffrey M. Kidd, Ryan E. Mills

**Affiliations:** Department of Computational Medicine & Bioinformatics, University of Michigan, Ann Arbor, MI 48109 USA; Department of Human Genetics, University of Michigan, Ann Arbor, MI 48109 USA

**Keywords:** Structural variation (SV), Complex structural rearrangements, Sequence analysis, Human, Copy number variant (CNV)

## Abstract

Complex chromosomal rearrangements are structural genomic alterations involving multiple instances of deletions, duplications, inversions, or translocations that co-occur either on the same chromosome or represent different overlapping events on homologous chromosomes. We present SVelter, an algorithm that identifies regions of the genome suspected to harbor a complex event and then resolves the structure by iteratively rearranging the local genome structure, in a randomized fashion, with each structure scored against characteristics of the observed sequencing data. SVelter is able to accurately reconstruct complex chromosomal rearrangements when compared to well-characterized genomes that have been deeply sequenced with both short and long reads.

**Electronic supplementary material:**

The online version of this article (doi:10.1186/s13059-016-0993-1) contains supplementary material, which is available to authorized users.

## Background

Structural variation (SV), defined as chromosomal rearrangements resulting from the removal, insertion, or rearrangement of genomic regions, are natural sources of genetic variation [1–3] that have also been implicated in numerous human diseases [4–6]. There have been extensive studies to discover these genomic aberrations from the whole genomes of humans and other species and numerous algorithms have been developed to accurately identify their prevalence [7–11]. These approaches have primarily focused on simple copy number variants (CNVs; deletions, duplications) or copy neutral (inversions) rearrangements defined by at most two chromosomal breakpoints (BPs) and work by identifying and clustering various signals of discordant alignments from paired-end next generation sequencing data [12]. Recent algorithms have begun to integrate signals across multiple features to increase sensitivity [9, 11, 13] and these have been successful in precisely identifying various types of SVs. Knowledge of the underlying structure of the rearrangement is still required, however, in order to properly model these aberrant signals to the correct type of structural variant. For example, clusters of read pairs (RPs) with insert sizes (ISs) larger than expected are typically representative of deleted sequence since this observation is consistent with how the reads would map in the presence of such an event.

**Fig. 1:**
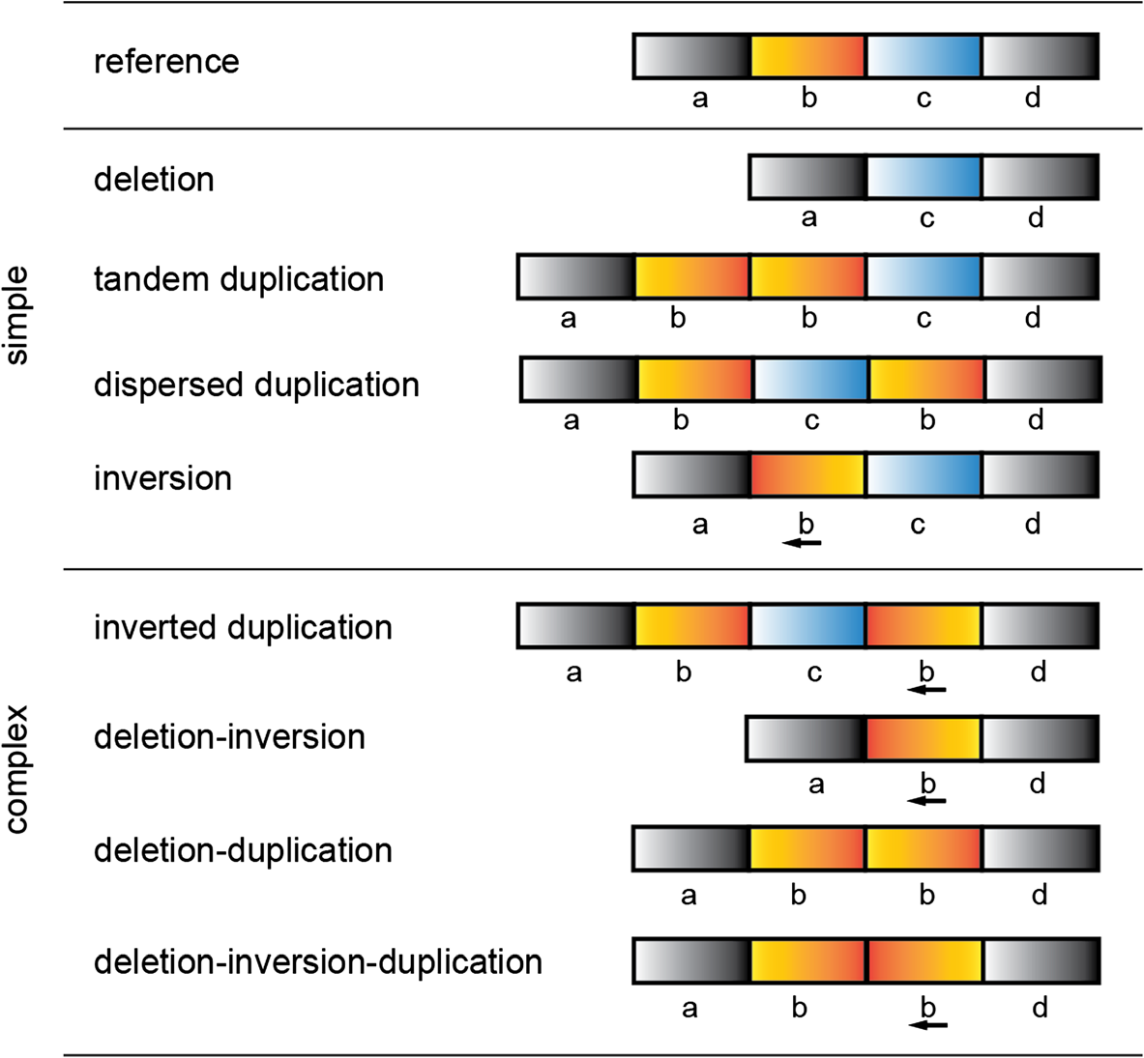
Illustration of simple and complex rearrangements, as compared to an unaltered reference genome. Simple rearrangements are typically defined by two BPs, although dispersed duplications include an additional BP at the insertion site. Examples of commonly observed complex structural variants with three or more BPs are provided but are non-inclusive

While these simple rearrangements are common in the genome, there are additional rearrangements that, while rarer, are far more convoluted. These complex SVs (CSVs) are typically represented by three or more BPs and can arise from a variety of mechanisms including fork stalling and template switching (FoSTeS) and microhomology-mediated break-induced replication (MMBIR) (Fig. 1, reviewed in [14]). Although fairly common in cancers, their prevalence in germline genomes is gradually becoming more apparent as is their potential role in the pathogenesis of other disease [4, 5, 15]. The complex nature of these events have made them challenging to accurately detect and catalog and many CSVs have been either neglected or misinterpreted by current techniques due to the complexity of the signals shown by the sequencing data. This is primarily due to the limitations of presupposing the types of SVs being considered, as oftentimes the signals from one event are clustered independently from those of another and can lead to contradictory or sometimes even opposing predictions to what is actually present. Under such circumstances, traditional prediction models lose their ability to discriminate between signals and therefore new computational strategies are required to overcome these challenges. Previous endeavors have been made to reconstruct somatic variants in cancer genomes both spatially [16, 17] and temporally [18], but require an unaltered “matched” germline genome as an anchor for comparison. Studies into CSVs in the germline itself to date have thus been more limited, though there has been some early work that has profiled the existence of some of the more common types of CSVs including inverted-duplications and deletion-inversions [19].

Here, we present a novel approach, SVelter, to accurately resolve complex structural genomic rearrangements in whole genomes. Unlike previous “bottom up” strategies that search for deviant signals to infer structural changes, our “top down” approach works by virtually rearranging segments of the genomes in a randomized fashion and attempting to minimize such aberrations relative to the observed characteristics of the sequence data. In this manner, SVelter is able to interrogate many different types of rearrangements, including multi-deletion and duplication-inversion-deletion events as well as distinct overlapping variants on homologous chromosomes. The framework is provided as a publicly available software package that is available online (https://github.com/mills-lab/svelter).

## Results

### Overview of SVelter

**Fig. 2:**
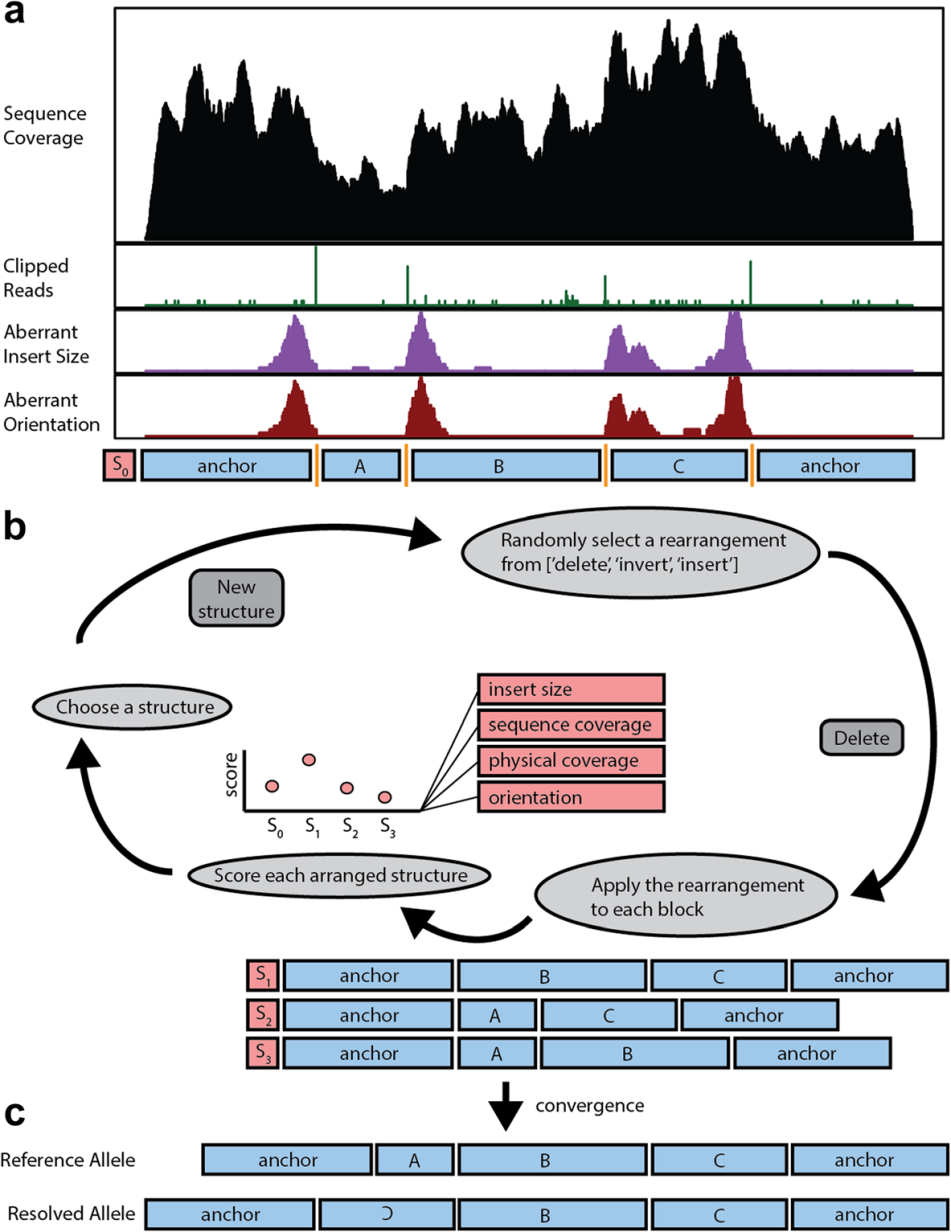
Overview of computational strategy for identifying SV in whole genome sequences. **a** SVelter first scans the genome and identifies clusters of aberrant read characteristics. These are used to create a putative set of BP positions. **b** The segments between BPs are then iteratively rearranged and scored against null models of sequence expectations. **c** The final converged structure is reported as the predicted structural rearrangement for the region

Our approach predicts the underlying structure of a genomic region through a two-step process. SVelter first identifies and clusters BPs defined by aberrant groups of reads that are linked across potentially related structural events. It then searches through candidate rearrangements using a randomized iterative process by which rearranged structures are randomly proposed and scored by statistical models of expected sequence characteristics (Fig. 2; see “Methods”). In this fashion, it resembles a Gibbs sampling approach as has been previously utilized for motif finding [20] and haplotype reconstruction using single nucleotide polymorphisms (SNPs) [21], among other applications.

SVelter begins by fitting statistical models for IS and read depth (RD) based on paired-end sequences sampled from copy neutral genomic regions [22]. Both are modeled as normal distributions for efficiency purposes which is recommended for relatively clean data sequenced at higher depth; however, more accurate but slower models (i.e. binomial for IS and negative binomial for RD) are also available as options for data of lower quality. SVelter then searches for and integrates potential SV signals from RPs with aberrant IS, orientation, and/or alignment (soft-clipping). Pairs of BPs are assigned simultaneously and BP pairs that intersect with each other are further connected to form BP clusters. For each cluster containing *n* BPs, the *n-1* genomic segments defined by adjacent BPs are then rearranged in a randomized iterative process whereby a simple SV (deletion, insertion, inversion) is randomly proposed and applied to all possible segments to assess the viability of each putative change. The initial structure and each subsequent rearranged structure are then scored by examining the impact of each change on various features of the sequence reads in the region, including IS distribution, sequence coverage, physical coverage, and the relative orientation of the reads. A new structure is then chosen for the next iteration using a probability distribution generated from the structure scores. This continues until the algorithm converges on a final, stable set of rearrangements or a maximum number of iterations is reached.

An important feature of SVelter is that it simultaneously constructs and iterates over two structures, consistent with the zygosity of the human genome. This allows for the proper linking of BP segments on the correct haplotypes, which is crucial for the proper resolution of overlapping structural changes that can often confuse or mislead other approaches. Individual breaks in the genome can then be properly linked and segregated, aiding in downstream genotyping across multiple individual sequences.

The randomized aspect of this approach leads to additional computation cost relative to other SV detection algorithms. We have addressed this by implementing a number of optimizations to increase the overall efficiency of SVelter. First, we limit the number of clustered BPs during the initial BP-linking step in order to manage the number of random combinations at the next step. For regions with potentially higher numbers of linked BPs, we form subgroups based on physical distance between adjacent BPs that are later combined. Second, we set an upper and lower bound on the potential copy number (CN) of each segment between BPs using local RD information and only allow structures containing *CN-1* to *CN + 1* blocks for downstream analysis. This results in a total processing time for SVelter on a re-sequenced human genome with 50X coverage of under 8 h when run in parallel on a high-performance computing cluster made up of Dell C6100 machines using 24 cores consisting of 2.67 GHz Intel Xeon X5650 processors, each with an allocated 8 GB of memory.

Another limitation due to the stochastic nature of this approach is that SVelter by default is primarily heuristic rather than rigorous. Thus, it is not only non-deterministic but can neither guarantee the optimality of its converged structures nor that every possible solution/structure was visited. A brute force method that interrogates every potential structure would address these issues but would be computationally prohibitive, especially for more complex rearrangements with a larger number of BPs and thus possible structures that would need to be permutated. We have attempted to balance SVelter in this regard by implementing a two-pass system where, after converging on a stable rearrangement for 100 continuous iterations, we set this structure aside and restart the random iterations for another 100 iterations, at which point the highest scoring structure overall is chosen. We also provide a deterministic option that is non-random and uses hill climbing to incrementally choose the best scoring structure, though we note that this will likely result in suboptimal results as the converged structure could represent a local rather than global minimum deviation in score from the null models.

### Performance evaluation

We compared SVelter to four SV detection algorithms: Delly [11], Lumpy [9], Pindel [8], and ERDS [23]. Both Delly and Lumpy have integrated IS and split read information into their SV detection strategy, while Pindel implements a pattern grown approach to utilize split read alignments. ERDS uses an integrative model that combines IS, RD, and SNP allele frequency to detect CN imbalances. While there are numerous other algorithms that have been developed for detecting SVs, we focused on these as they have previously published comparisons that can be transitively applied to our results.

Multiple experiments were conducted in order to evaluate our approach. We first simulated genomes of various sequence coverage containing both simple and complex SVs as homozygous and heterozygous events. We next applied these algorithms to the genome of a haploid hydatidiform mole (CHM1) [8, 24, 25] and also a well-characterized diploid genome (NA12878) [26, 27], both of which had reported high-confidence calls as well as long-read Pacific Biosciences (PacBio) sequences available for orthogonal assessment. All algorithms were run either with the recommended settings as provided by the authors (where available) or default settings. Detailed commands for running each algorithm can be found in Additional file 2.

#### Simulated data

**Fig. 3:**
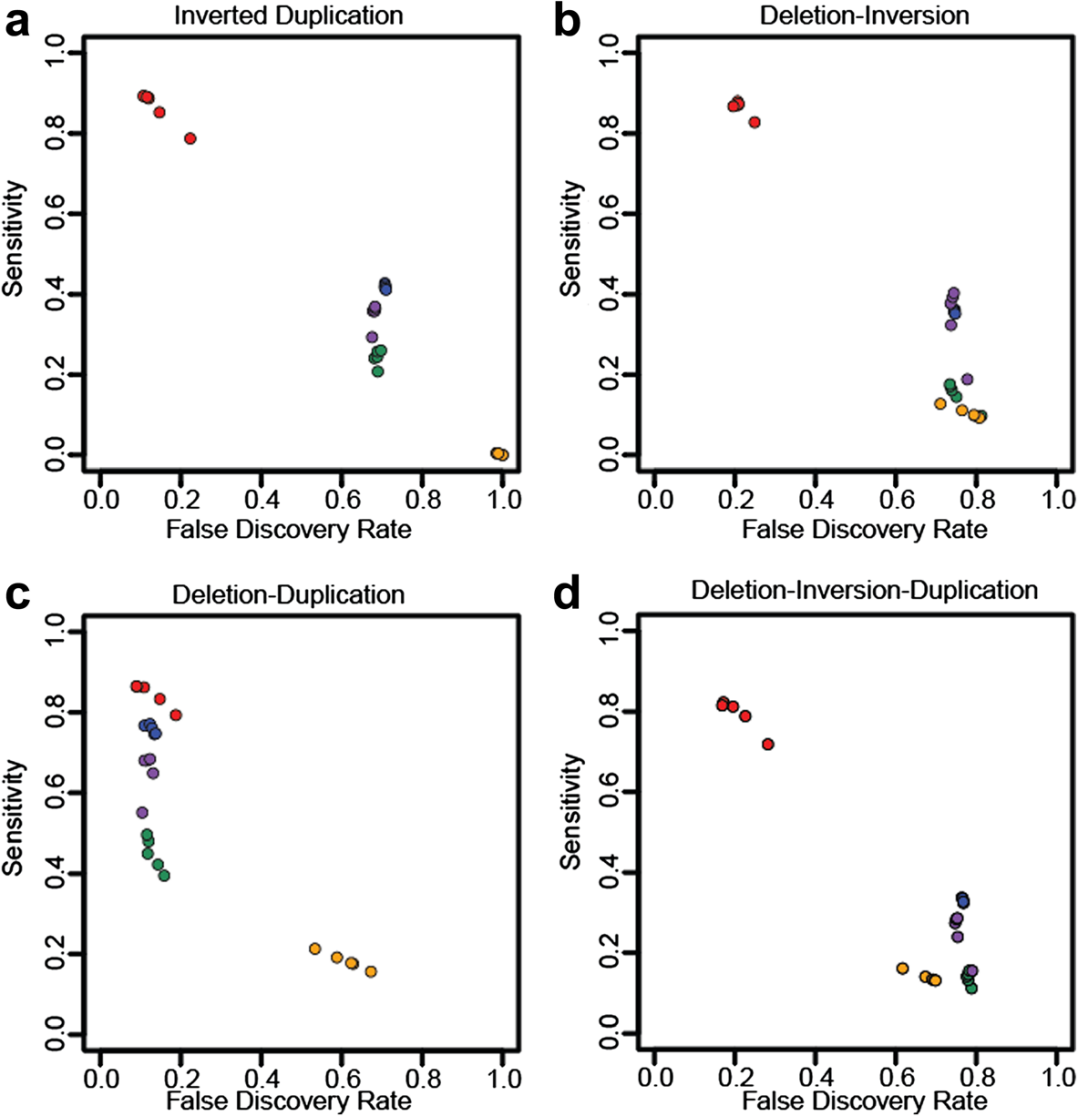
Assessment of CSV accuracy using simulated datasets. Sensitivity and FDRs for SVelter (*red*), Delly (*blue*), Lumpy (*green*), Pindel (*purple*), and ERDS (*yellow*) on simulated **a** inverted duplications, **b** deletion inversions, **c** deletion duplications, and **d** deletion-inversion-duplication events

We simulated specific types of complex rearrangements based on structures recently reported [28] as well as our own observations (Additional file 1: Table S1). Performance comparisons with complex structures are less straightforward than with simple SVs as most algorithms are only designed to identify simple events, and therefore may predict portions of CSVs as independent events. We address this issue by considering the identification and predicted CN of individual junctions as reported in the entire prediction set of each algorithm (deletions, duplications, inversions) and compared against each simulated complex event collectively, treating predicted non-simulated junctions in the region as false positives (FPs) (see “Methods”). SVelter performs consistently better in terms of sensitivity and false discovery rate (FDR) across almost all types of complex events (Fig. 3).

We also simulated heterozygous and homozygous non-overlapping simple SVs (deletions, inversions, tandem duplications, dispersed duplications, and translocations) of varied sizes into synthetic genomes sequenced at different depths of coverage (10–50X). We then calculated the sensitivity and FDR of each algorithm (Additional file 2: Figures S1–S3). Overall, SVelter achieves a higher sensitivity and lower FDR for simple deletions compared to all other algorithms. Comparisons with duplications were more difficult; while all compared approaches can report tandem duplications, for dispersed duplications only SVelter reports both the duplicated sequence and its distal insertion point. We therefore took a conservative approach such that for calculating sensitivity we compared the full set of duplications predicted from each approach to the simulated set of tandem and dispersed events, but limited the FP analysis to only tandem duplications for the other algorithms. It should be noted that this method of comparison would bias against SVelter to some extent; however, under these circumstances SVelter still showed very good sensitivity and positive predictive value in calling dispersed duplications, with slightly worse performance for tandem duplications. For inversions, SVelter showed a comparable accuracy to other the algorithms.

#### Real data

**Fig. 4:**
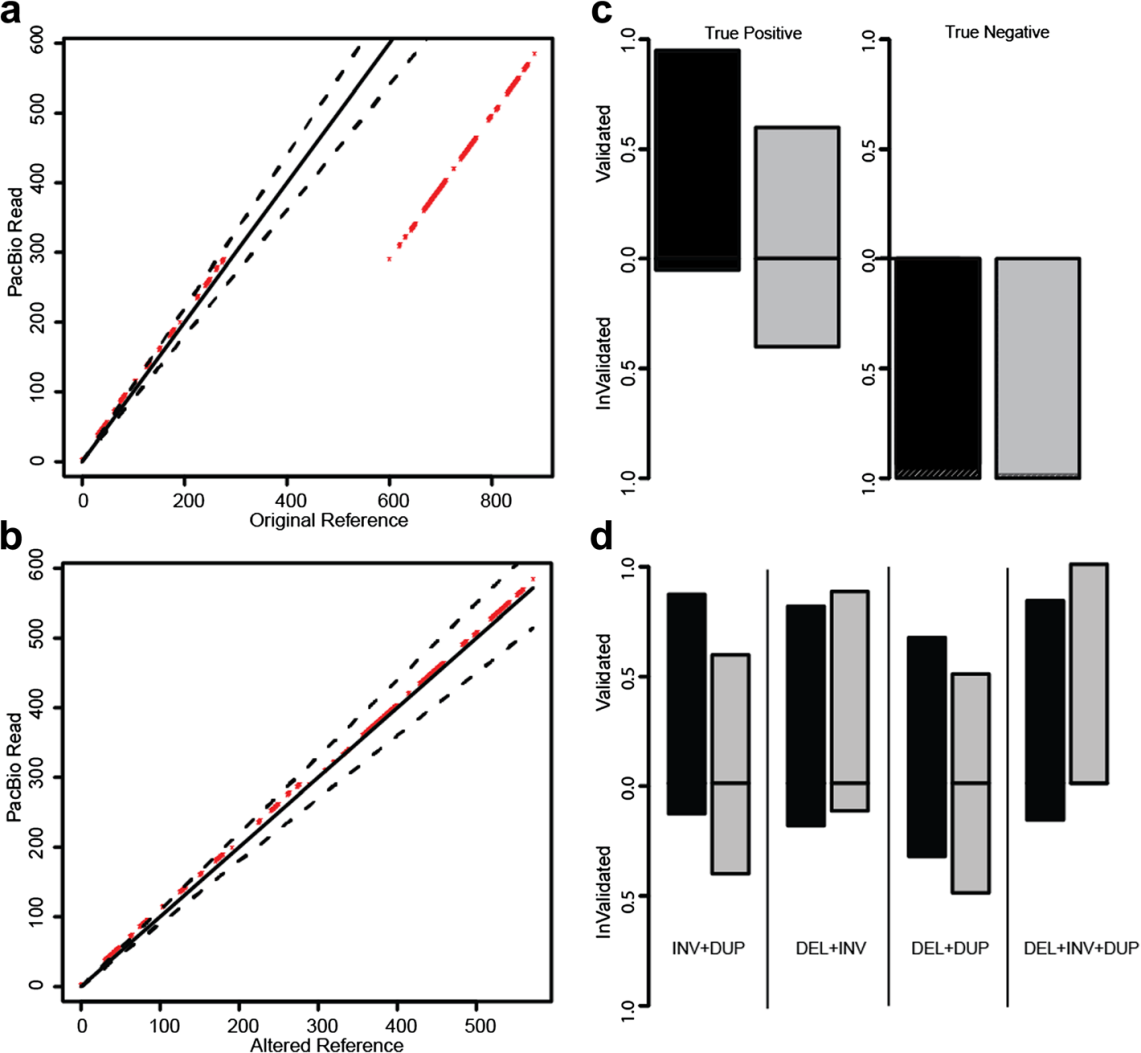
Overview and application of PacBio validation approach to human data. **a** *Dot plot* of the sample region containing a simple deletion using a single PacBio read against the reference genome. *Red dots* indicate matches between sequences and *dashed black lines* delineate 10 % deviance from the diagonal. **b** *Dot plot* of the same region using an altered reference incorporating the deletion event. **c** Fraction of TP calls using validation approach on published deletions in NA12878 (*black*) and CHM1 (*gray*) and CN2 regions as negative controls. *Dashed black lines* indicate regions that could not be assessed due to lack of PacBio reads to interrogate. **d** Assessment of specific predicted complex structures by SVelter using PacBio reads in NA12878 (*black*) and CHM1 (*gray*)

To evaluate how SVelter performs on real data, we have applied each algorithm to two publicly available datasets: CHM1 [24] and a well-characterized diploid genome analyzed by the Genome in a Bottle (GIAB) Consortium (NA12878) [26, 27]. Both have been deep sequenced by Illumina short-insert and PacBio long-read sequencing and provide excellent sets of benchmarking variants for simple SVs. However, there are few complex rearrangements annotated in either genome and this precluded a direct comparison with our results. We therefore examined the PacBio data directly for each predicted variant using a custom validation approach that utilizes a recurrence strategy to compare each read to both the reference allele as well as a rearranged reference consistent with the predicted structure (Fig. 4a, b, see “Methods”). We conducted PCR experiments on the predicted BPs of three predicted complex rearrangements that were validated with this approach to show convincing evidence for two, with inconclusive results for the third due to high degrees of repetitiveness in the region (Additional file 2: Figures S4–S7). We also evaluated this approach using sets of reported deletions in these samples as well as matched random sets located within copy neutral regions and found it to have very high true positive (TP) and true negative rates (Fig. 4c). We then assessed our approach on specific types of complex rearrangements in CHM1 and showed SVelter to have an overall high validation rate (Fig. 4d). We also observed an increase in accuracy on simple deletion calls across all algorithms after the application of our validation scheme (Additional file 2: Table S2).

**Fig. 5:**
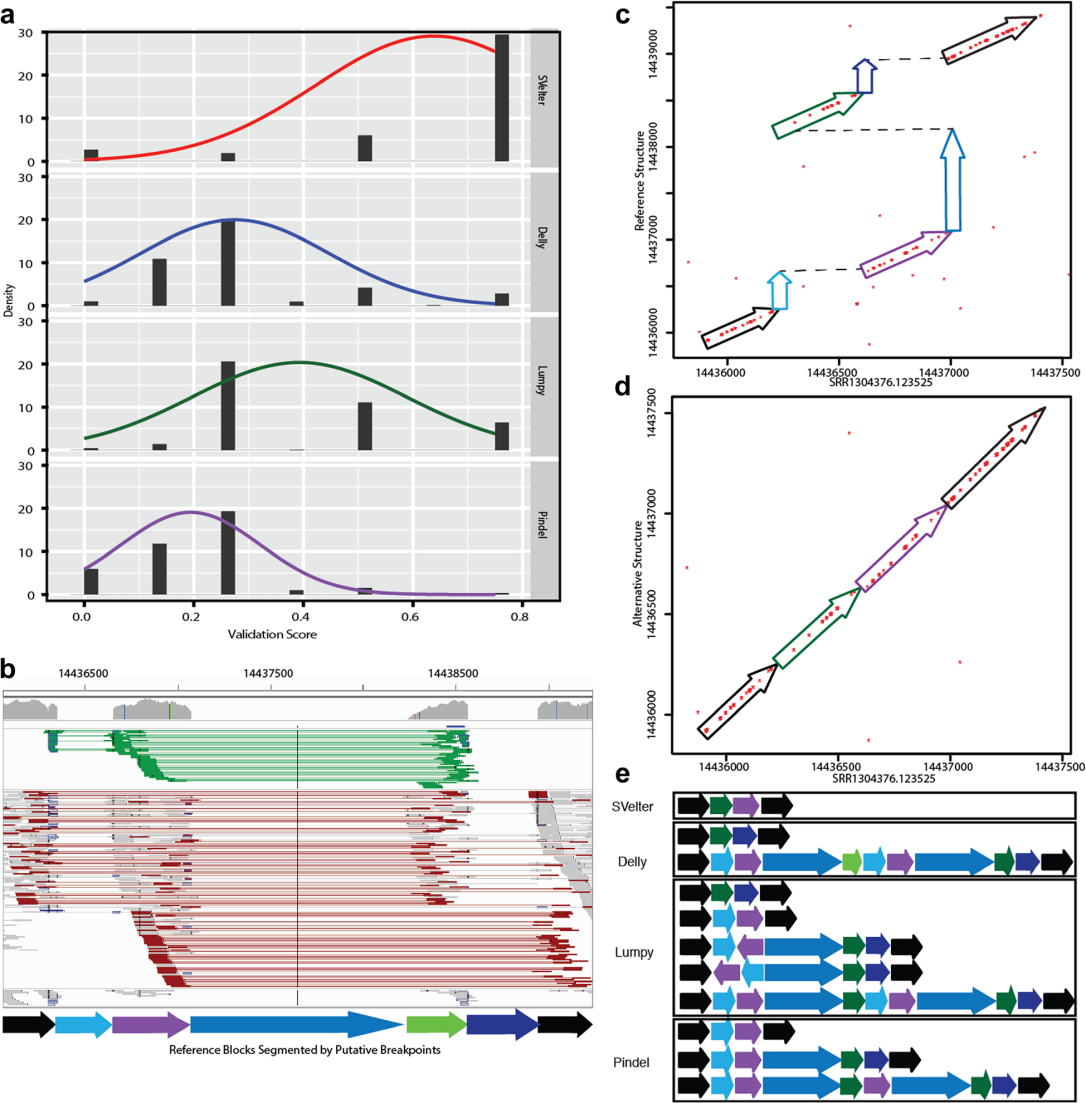
Evaluation of CSV predictions. **a** Validation scores of CSV predicted in NA12878 from all algorithms ranked and normalized from 0 to 1 for comparison. For approaches with multiple predicted SVs in a region, average scores from each prediction were averaged. **b** *IGV screenshot* of example complex region in CHM1 (chr1:14435000-1444000) containing multiple deletions (*blue shaded arrows*) and a translocated region (*green arrow*), with surrounding anchor regions in *black. Light green lines* in IGV indicate RPs with reverse-forward orientation, while *red lines* indicate RPs with aberrant IS length. **c** *Dot plot* of region between an individual PacBio read (SRR1304376.123525) against the reference sequence. *Colored arrows* correspond to segments indicated in (**b**). **d** *Dot plot* of altered reference sequence implementing predicted rearrangements by SVelter. **e** *Schematic* of predictions by each SV algorithm with respect to segments indicated in (**b**). For approaches with multiple predictions overlapping the region, each predicted SV is shown independently

We then compared the performance of each algorithm on identifying and resolving CSVs. Given that there are very few reference sets available of known complex rearrangements, we first created a set of non-overlapping candidate CSVs as identified by SVelter in CHM1 and NA12878. We then collected all predictions from each algorithm that overlap that region and scored them using the validation approach above. As many complex rearrangements may be described as a combination of simple SVs, we utilized a ranking approach to compare the individual predictions by assigning 0 to the lowest scores and 0.75 to the highest scores (see “Methods”). We observed a significant enrichment of SVelter predictions with high validation scores, indicative of its efficacy in correctly resolving CSVs (Fig. 5a). An example is shown in Fig. 5b, which depicts a summary of sequence read alignments for a region on chromosome 1 in CHM1 containing multiple deletions as well as a local translocation. Using standard read clustering algorithms, the signals present might suggest the presence of a tandem duplication overlapping with large deletions. However, this is not consistent with the haploid nature of CHM1 and comparisons with long PacBio sequence reads that overlap the region show the true structure (Fig. 5c), which when aligned to a rearranged reference using SVelter predictions shows a full length alignment (Fig. 5d). A comparison with other algorithms indicates that their predictions are indeed consistent with analyzing each aberrant read cluster independently of each other and result in a combination of tandem duplications, deletions, and inversions (Fig. 5e).

#### Computational runtime

We compared the overall executable runtime of the different software packages using a single chromosome from NA12878. For each algorithm, we initialized the analysis using a previously aligned sequence in BAM format and used the respective procedures necessary for each approach to result in a variant call file (see “Methods”). Delly was observed to complete the fastest, followed by Lumpy. Pindel and SVelter were somewhat slower and were comparable in their runtime (Additional file 2: Table S3). It should be noted that some algorithms (e.g. Lumpy) can perform faster with optimized alignment strategies [29], however this was not included in our assessment.

### Examination of identified SVs in CHM1 and NA12878

**Fig. 6:**
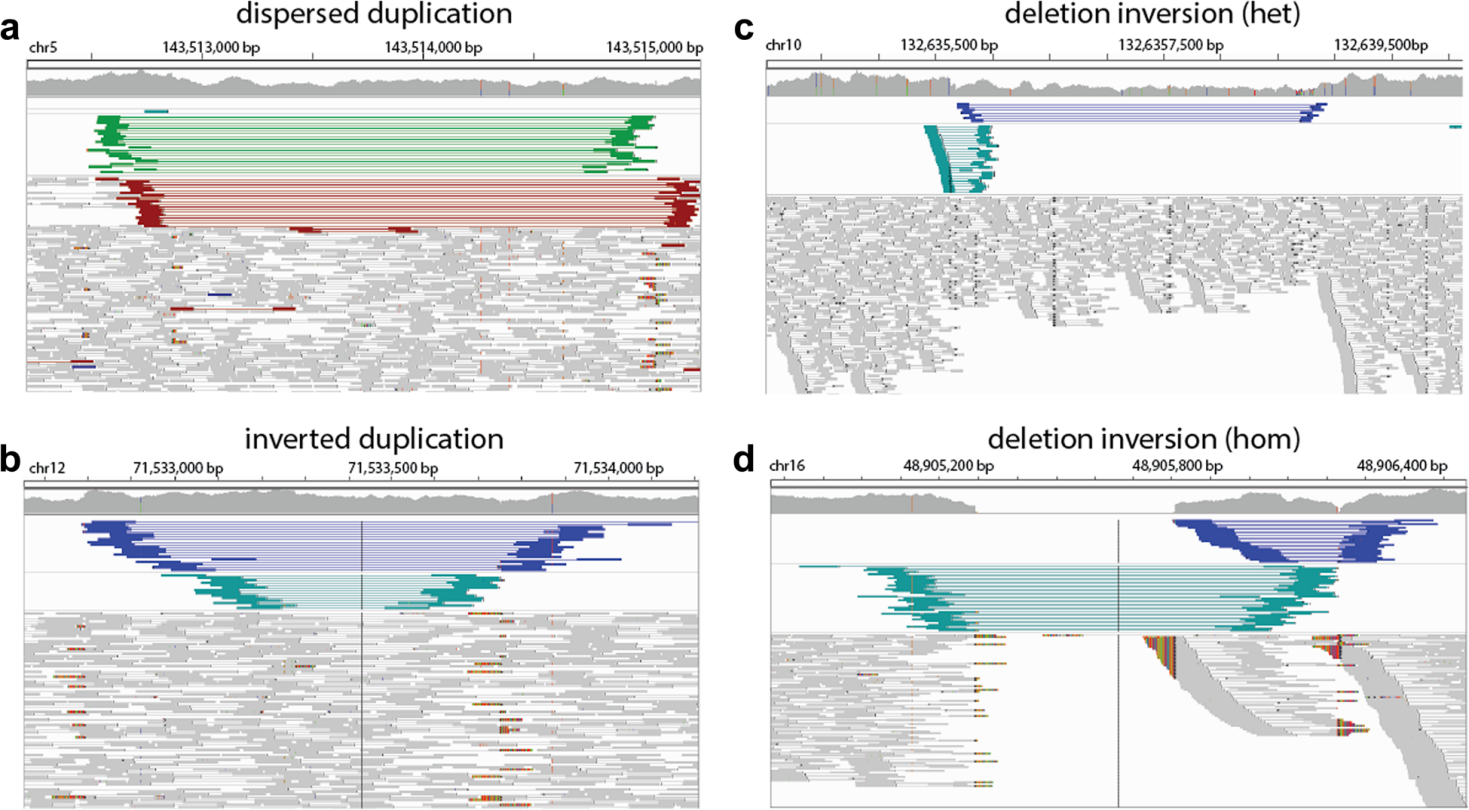
Examples of various types of CSV in NA12878 identified by SVelter. **a** *IGV screenshot* of disperse duplication event predicted by SVelter. *Line colors* as described in Fig. 4. Such regions are typically identified as an overlapping tandem duplication and deletion. **b** Example of inverted duplication event. *Blue lines* in IGV indicated reverse-reverse RP orientation while *dark green lines* indicate forward-forward RP orientation. **c** Region with heterozygous inversion and deletion rearrangement. **d** Region with homozygous inversion and deletion rearrangement. All regions shown had PacBio sequences consistent with predicted SVelter structures and were misclassified by other approaches (Additional file 2: Figures S8–S11)

We examined the full set of identified simple and complex SVs in both CHM1 and NA12878. As expected, we rediscovered many previously reported deletions, duplications, and inversions (Table 1). In some cases, we were also able to identify dispersed duplications that were incorrectly identified as overlapping tandem duplication and deletion events in prior reports (Fig. 6a, Additional file 2: Figure S8). Furthermore, we found a recurrence of particular types of CSVs, including inverted-duplication and deletion-inversion events (Fig. 6b–d, Additional file 2: Figures S9–S11) suggesting that they are likely more common than previously thought. However, there were numerous other CSVs that could not be coalesced into a single classification and may provide future insight into new mechanisms for SV formation.

**Table 1:** Predicted SV types in CHM1 and NA12878 by SVelter

SV type	CHM1	NA12878
Simple DEL	1003 (0.72)	1867 (0.95)
Simple DUP	897 (0.61)	790 (0.61)
Tandem	834 (0.62)	755 (0.60)
Dispersed	63 (0.56)	35 (0.71)
Simple INV	48 (0.75)	107 (0.76)
Simple TRA	24 (0.67)	30 (0.83)
INV + DUP	126 (0.59)	29 (0.86)
DEL + INV	8 (0.88)	26 (0.81)
DEL + DUP	8 (0.50)	12 (0.67)
DEL + DUP + INV	2 (1.00)	6 (0.83)
Other	112 (0.63)	204 (0.77)

Numbers in parenthesis indicate percentage of calls with PacBio validation support. The remaining calls either were not able to be assayed with our approach or were invalidated

## Discussion

We have described an integrative approach, SVelter, that can identify both simple and complex structural variants through an iterative randomization process. We show that it has an improved or comparable accuracy to other algorithms when detecting deletions, duplications, and inversions but has the additional capability of correctly interpreting and resolving more complex genomic rearrangements with three or more BPs. Furthermore, SVelter can resolve structural changes on parental haplotypes individually, allowing for the correct stratification of multiple overlapping SVs. SVelter achieves this by forgoing the assumption of specific patterns of read alignment aberrations as associated with individual rearrangements and instead allowing the underlying sequence itself to dictate the most probable structure.

The ability to accurately identify CSVs in whole genome sequence data is a significant advancement, as currently many such regions are either missed or identified as individual errant events. For example, in our investigation of NA12878 we identified many disperse duplications that were previously reported as overlapping deletion and tandem duplication events as well as other simple deletions and inversions that were in fact part of a larger complex rearrangement (Fig. 5). Such regions could be, in part, responsible for the observed discrepancies when comparing different SV algorithms with each other as well as other platforms such as array-CGH [30]. Our observations are also consistent with recent findings by the 1000 Genomes Project [28], however their analysis required the use of multiple long-read sequencing technologies including PacBio and Moleculo to interpret the regions while SVelter is able to correctly resolve the regions from short-insert Illumina sequences alone. Although long-read technologies are very well suited for such an application, their use is currently limited to small-scale projects and there have been estimates that over 300,000 genomes have been sequenced using Illumina short-insert reads in 2015 alone. Thus, approaches like SVelter that perform accurately on such datasets are likely to have a larger impact on correctly reporting complex structural genomic aberrations, though they will have lesser ability to detect and reconstruct novel insertion sequences compared to long-read approaches.

While SVelter was specifically designed to identify and resolve complex rearrangements, it also surprisingly showed a slight increase in accuracy in identifying certain types of simple SVs when compared to other modern approaches. One potential factor that may contribute to this observation is that SVelter determines the presence of an SV in a quantitative and not qualitative manner. Specifically, most other paired-end algorithms typically utilize a standard deviation-based cutoff to determine whether the observed IS fragments are larger than would be expected from the constructed library; thus, two paired sequence reads are either aberrant or normal. SVelter, however, scores each observation directly from the IS probability density function. For example, for an IS library with mean = 350, an observation of 375 will score better than one of 475, even if both are within 3 standard deviations of the overall IS distribution. When combined with the signals of RD and physical coverage over potential BPs, we feel this adds additional granularity for identifying SVs, particularly for smaller (<1 kb) events.

One limitation of SVelter is that, even with our efficiency enhancements, it still exhibits a longer processing time with respect to the other SV algorithms compared here. This is in part due to the randomization strategy but is also owing to the inclusion of a read coverage component, which is not modeled in the other approaches we compared against but contributes to the overall increased accuracy of SVelter. Recent advances have made it possible to analyze a high coverage human genome from sequence to variant calling and annotation in half a day [29] and such applications are very useful for diagnostic applications where speed is a critical component. Nevertheless, the enhanced ability of SVelter to correctly resolve overlapping and complex rearrangements relative to other approaches makes it very useful for projects where the accurate detection of such regions is important. Another limitation of SVelter is that in its current form it has a reduced ability to delineate heterogeneous data, such as commonly found when sequencing cancer genomes. This is due to its expectation of a specific ploidy when iterating between multiple haplotypes. Future work in this area will focus on creating a dynamic structure that can allow different levels of heterogeneity or mosaicism.

## Conclusions

We have developed and applied a new approach to accurately detect and correctly interpret both simple and complex structural genomic rearrangements. Our comparisons to existing algorithms and datasets show that SVelter is very well suited to identifying all forms of balanced and unbalanced SV in whole genome sequencing datasets.

## Methods

### SVelter algorithm

SVelter takes aligned Illumina paired-end sequence data in sorted BAM format as input as well as the reference genome against which the sequences were aligned and reports all predicted SVs in both a custom format as well as VCFv4.1. Default parameters are chosen to best balance sensitivity and efficiency, though are adjustable for users to best fit their own data. The SVelter framework consists of three major modules: null model determination, BP detection, random iterative rearrangement, and structure scoring (Fig. 2).

#### Null model determination

SVelter first filters the reference genome to exclude regions of low mappability from downstream analysis to increase efficiency by avoiding regions where alignments are unreliable. Such regions include gaps and unknown regions in the reference genome (Ns) and these are integrated with previously reported genomic regions identified by ENCODE [31] (wgEncodeDacMapabilityConsensusExcludable and DukeMapabilityRegionsExcludable obtained from UCSC Genome Browser) that are of low mappability to form a final version of excluded regions. Next, the IS distribution (*f*_*IS*_) is determined by calculating the mean (*μ*_*IS*_) and standard deviation (*σ*_*IS*_^2^) of all RPs aligned to genomic regions that are either randomly sampled or collected from a set of copy neutral (CN2) genomic regions defined as places in the genome where no polymorphic CNVs, segmental duplications, or repetitive elements have been annotated and thus providing a good estimate of the baseline alignment characteristics [22]. Normal distribution is constructed (*f*_*IS*_ ∼ *N*(*μ*_*IS*_, *σ*_*IS*_^2^)). A normal distribution of RD (*f*_*RD*_ ∼ *N*(*μ*_*RD*_, *σ*_*RD*_^2^)) and physical coverage (*f*_*PC*_ ∼ *N*(*μ*_*PC*_, *σ*_*PC*_^2^)) are characterized by sliding a fixed-size window (default: 100 bp) across the same genomic region and constructing the sample mean and standard deviation. Alternatively, in situations where the RD is not high enough be approximated as normal (empirically, <10X), SVelter provides options for more complex but less efficient models, i.e. bimodal (fitted by *mixtools*) for IS,

$$ {f}_{IS}\sim p\times N\left({\mu}_{I{S}_1},{\sigma}_{I{S}_1}^2\right)+\left(1-p\right)\times N\left({\mu}_{I{S}_2},{\sigma}_{I{S}_2}^2\right) $$

and negative binomial for RD and physical coverage:

$$ \begin{array}{l}{f}_{RD}\sim NB\left({r}_{RD},{p}_{RD}\right),\  where\ {r}_{RD}=\frac{{\mu_{RD}}^2}{{\sigma_{RD}}^2-{\mu}_{RD}},\ {P}_{RD}=1-\frac{\mu_{RD}}{{\sigma_{RD}}^2}\hfill \\ {}{f}_{PC}\sim NB\left({r}_{PC},{p}_{PC}\right), where\ {r}_{PC}=\frac{{\mu_{PC}}^2}{{\sigma_{PC}}^2-{\mu}_{PC}},\ {P}_{PC}=1-\frac{\mu_{PC}}{{\sigma_{PC}}^2}\hfill \end{array} $$

#### Detection and clustering of putative BPs

SVelter next scans the input alignment file to define putative BPs where the sample genome differs from the reference. These are defined through the identification of aberrant read alignments. Clusters of RPs showing abnormal insert length or aberrant mapping orientation may indicate BPs nearby, while reads with truncated (clipped) split read (SR) alignments are indicative of precise BP positions. SVelter specifically defines aberrant reads as follows:

1. RPs outside expected IS (*μ*_*IS*_ ± *s* × *σ*_*IS*_, where *s* is the number of standard deviation from the mean, default as 3);
2. RPs that do not have forward reverse pair orientation;
3. SRs with high average base quality (i.e. 20) clipped portion with minimum size fraction of overall read length (i.e. 10 %).

It should be noted that the default parameters used by SVelter were determined empirically and can be adjusted by the user. Discordant RPs of the within a window of mean IS + 2*std distance and of the same orientation are clustered together. Next, split reads within this window and downstream of the read direction are collated and the clipped position is considered as a putative BP. If no such reads exist, the rightmost site of forward read clusters or leftmost site of reverse read clusters is assigned instead. For each cluster of aberrant RPs, a BP is assigned if the total number of split reads exceeds 20 % of the RD or the total number of all aberrant reads exceeds 30 %. For samples of poorer quality, higher cutoffs might be preferred. Each putative BP will be paired with other BPs that are defined by mates of its supporting reads. BP pairs that intersect or are physically close (<1 kb) to each other will be further grouped and reported as a BP cluster for the next step.

#### Random iterative rearrangement

For each BP cluster containing n putative BPs, a randomized iterative procedure is then applied on the n-1 genomic blocks between adjacent BPs. SVelter has three different modules implemented for this step: diploid module (default) that detects structural variants on both alleles simultaneous, heterozygous module that only report high quality heterozygous SVs, and homozygous module for high quality homozygous SVs only. For the diploid module, a simple rearrangement (deletion, inversion, or insertion) is randomly proposed and applied to each block on one allele while the other allele is kept unchanged and the newly formed structure is scored against the null models of expectation for each feature through the scoring scheme described below. A new structure is then selected probabilistically from the distribution of scores such that higher scores are more likely but not assured. The same approach is then applied to the other allelic structure representing a single iteration overall. For heterozygous and homozygous modules, only one allele is iteratively rearranged while the other allele remains either unchanged or is mirrored, respectively.

The iterative process will terminate and report a final rearranged structure if one of the following configurable situations is met:

1. No changes to a structure after 100 continuous iterations; or
2. The maximum number of iterations is reached (100,000 as default).

After the initial termination, the structure is reset and the process is repeated for another 100 iterations while avoiding the fixed structure, at which point the highest scoring structure overall is chosen.

#### Structure scoring

Assume *S*_*j*_ as the score of rearranged structure *j*. To estimate *S*_*j*_, four different characteristics of RP *i*: IS (*IS*_*ij*_), Pair Orientation (*PO*_*ij*_), RD (*RD*_*ij*_), and Physical Coverage Through a BP (*PC*_*ij*_) would be calculated and integrated. As the distribution of IS, RD, and Physical Coverage has been defined, the density function would be calculated and transformed to log scale:

$$ \begin{array}{l} Score\_I{S}_{ij}= log\left({f}_{IS\ }\left(I{S}_{ij}\right)\right)\hfill \\ {} Score\_R{D}_{ij}= log\left({f}_{RD}\left(R{D}_{ij}\right)\right)\hfill \\ {} Score\_P{C}_{ij}= log\left({f}_{PC}\left(P{C}_{ij}\right)\right)\hfill \end{array} $$

Score of Pair Orientation is specified by the indicator function:

$$ Score\_P{O}_{ij}=\left\{{}_{0,\  if\  other\  wise}^{1,\  if\ PO= Forward - Reverse}\right\} $$

Assuming total number of n pairs of reads are aligned in the targeted genomic region, for each structure j, individual scores of each RP would be integrated to form the structure score:

$$ {S}_i={\displaystyle \sum_{i=1}^n}\  Score\_I{S}_{ij}\times \left(1+\frac{{\displaystyle {\sum}_{i=1}^n}\  Score\_P{O}_{ij}}{n}\right)+\tau {\displaystyle \sum_{i=1}^n} Score\_R{D}_{ij}\times \left(1-{\displaystyle \sum_{i=1}^n}\  Score\_P{C}_{ij}\right) $$

where $\tau =\frac{log\left({f}_{IS}\left({\mu}_{IS}\right)\right)}{log\left({f}_{RD}\left({\mu}_{RD}\right)\right)}$ serves as the factor to regulate two parts into same scale.

### Performance assessment

Both simulated and real data were used to evaluate performance of SVelter. To produce simulation datasets, we altered the human GRCh37 reference genome to include both homozygous and heterozygous simple SVs and complex SVs independently while adding micro-insertions and short tandem repeats around the junctions in frequencies consistent with previously reported BP characteristics [32]. Details about specific types of SVs simulated are summarized in Additional file 1: Table S1, and specific details regarding the generation of the simulated data can be found in Additional file 3: Supplemental Methods. Paired-end reads of 101 bp with an IS of 500 bp mean and 50 bp standard deviation were simulated using wgsim (https://github.com/lh3/wgsim) across different RDs (10X, 20X, 30X, 40X, 50X).

For comparisons using real sequence data, we adopted two previously published samples: CHM1 [24] and NA12878 [18]. CHM1 has been deep sequenced by Illumina whole-genome sequence to 40X and by Single Molecule, Real-Time (SMRT) sequencing to 54X, and SVs of the sample have been detected and published by the same group as well (http://eichlerlab.gs.washington.edu/publications/chm1-structural-variation/). NA12878, together with the other 16 members from CEPH pedigree 1463, has been deep sequenced to 50X by Illumina HiSeq2000 system (http://www.illumina.com/platinumgenomes/). Additionally, the GIAB Consortium has published the PacBio sequencing data (20X) of NA12878 and also provided a set of high-confident SV calls [24, 27].

We assessed SVelter against four other algorithms with diverse approaches: Pindel, Delly, Lumpy, and ERDs. We applied these algorithms to both simulated and real data with default settings, except that SVelter’s homozygous module was used for CHM1. All algorithms were compared using the same set of excludable regions and were run on the same computing cluster.

#### Assessment of simulated simple SVs

For simulated datasets, we compared the performance of each algorithm by calculating their sensitivity and FDR on each type of simple SV (deletion, disperse duplication, tandem duplication, inversion). As Lumpy reports BPs in terms of range, we calculated the median coordinate of each reported interval and consider it as the BP for downstream comparison. A reported SV would be considered as a TP if the genomic region it spanned overlapped with a simulated SV of the same type by over 50 % reciprocally. As Delly and Lumpy did not differentiate tandem and dispersed duplication in their SV report, we compare their reported duplications to both simulated tandem and dispersed duplications independently to calculate sensitivity, but use the entire set of simulated duplications together for the calculation of specificity. In this manner, the result will be biased towards higher TP and TN rates for these approaches. Dispersed duplications reported by Pindel were very rare and as such were processed in the same way as Delly and Lumpy.

#### Assessment of real SVs

We initially made use of reported simple and complex SVs in CHM1 and NA12878 as gold standard sets; however, the FP rate of each algorithm was high compared to previously published performance. To augment this set, we therefore have developed our own approach to validate simple and complex SVs using PacBio long reads. For each reported SV, we collect all PacBio reads that go through the targeted region and hard clip each read prior to the start of the region. We then compare each read to the local reference and an altered reference reflecting the structure of the reported SV by sliding a 10 bp window through the PacBio read and aligning it against the reference sequence. Coordinates of each window are plotted against its aligned position in the form of a recurrence plot. If a read was sampled from the reference genome, most of the matched points should distribute close to the diagonal line. However, if a read was sampled from an altered genomic region, a continuous diagonal line would only show when plotted against a correctly resolved sequence. In this manner, shorter SVs can be validated by accessing the deviation of all matched points from diagonal. If aligning long read *j* against reference *k*, deviation of point *i* (*x*_*ijk*_, *y*_*ijk*_) is defined as *d*_*ijk*_ = |*x*_*ijk*_ − *y*_*ijk*_|, i.e. the vertical distance of the point to the diagonal. The deviation score of each *j* is calculated by summing up deviation of all points

$$ {S}_{jk} = {\displaystyle {\sum}_{i=1}^n{d}_{ijk}} $$

where *n* is the number of matches. For each long read *j*, the recurrence plot is made against the reference in both the original and altered formats, with corresponding scores *S*_*j*,*k* = *orig*_*and S*_*j*,*k* = *alt*_ assigned, and the score for the read is defined as

$$ {S}_j=\frac{S_{j,k= orig}}{S_{j,k= alt}}-1 $$

such that a positive score indicates the priority of altered genome over reference genome, and vice versa. The validation score of an SV is defined as proportion of supportive reads among the total *m* interrogated reads

$$ {S}_{val}=\frac{{\displaystyle {\sum}_{j=1}^m}\ I\left(1,\  if\ {S}_j > 0;\ 0,\  otherwise\right)\ }{m\left( number\  of\  reads\  checked\right)} $$

SVs with validation score >0.5 for haploid genome, or >0.3 for diploid genome would be considered validated. We further assess our ability to interrogate SVs in this fashion by scoring the reference sequence against itself at each region. In highly repetitive regions, the deviation scores will be higher overall and we can label such regions as non-assessable.

For longer (>5 kb) SVs, PacBio reads spanning through the whole targeted region are rarely observed in these data. In this situation, we scored each BP by adding 500 bp flanks and assessing each individually. The final validation score is then determined through the collation of matches from all BPs.

We reassessed our initial TP and FP simple calls from each algorithm by combining our PacBio validated SVs from each algorithm together with the reported calls. For simple SVs, we utilized a 50 % reciprocal overlap criterion. However, for CSVs we utilized a more complex comparison strategy to take into account that some algorithms will often detect individual parts of a complex rearrangement as distinct events. With each CSV predicted by SVelter, we extracted SVs with over 50 % reciprocal overlap from other algorithms and calculated the validation score for each of them using our PacBio validation approach described above. When multiple SVs were extracted from an algorithm, averaged scores were adopted. Validation scores of a CSV from all algorithms were ranked and normalized from 0 to 1 for comparison.

## Software and data availability

The software package *SVelter* (v1.1.2) is available for download at https://github.com/mills-lab/svelter as open source under the MIT License and additional documentation regarding specific software usage and parameters, supporting files, algorithm comparisons, and simulated datasets are provided at this site.

Simulated whole genome sequence data were generated as outlined in the supplemental code from synthetic reference sequences that can be obtained from https://umich.box.com/v/svelter.

Sequence data used in this analysis were obtained from the following resources:

CHM1 – Resolving the complexity of the human genome using single-molecule sequencing (http://eichlerlab.gs.washington.edu/publications/chm1-structural-variation/) [24].

NA12878 – Genome in a Bottle Consortium (https://sites.stanford.edu/abms/giab) [24, 26], Illumina Platinum Genomes (http://www.illumina.com/platinumgenomes/).

## Additional files

Additional file 1: Contains Supplemental Table 1 outlining the type and number of SVs included in each simulated genome and the stratified results for each algorithm. (XLSX 111 kb) Additional file 2: Contains Supplemental Tables 1–3 and Supplemental Figures 1–11 (DOCX 1461 kb) Additional file 3: Supplemental Methods outlining the software and parameter usage that was used to generate the presented results. (DOCX 10 kb)
