# Supplementary material for: Resolving complex structural genomic rearrangements using a randomized approach
Source: Genome Biol. 2016 Jun 10;17:126. doi: 10.1186/s13059-016-0993-1 (PMC4901421; doi:10.1186/s13059-016-0993-1)
Supplement: Additional file 2: — Contains Supplemental Tables 1–3 and Supplemental Figures 1–11 (DOCX 1461 kb) [file 13059_2016_993_MOESM2_ESM.docx]

Supplemental Table 1. Overview of results from application of each algorithm to SVs in simulated whole genome data sets across different sequence coverage and SV type.

(see Additional File 1)

Supplemental Table 2. True positive and false positive deletion calls made by each algorithm on NA12878 and CHM1, based on previous reported calls as well as our custom PacBio validation approach.

|  |  | Method | SV | TP | Ref  Calls | Total  Calls | FP | TPR | PPV | F1-Score |
| --- | --- | --- | --- | --- | --- | --- | --- | --- | --- | --- |
| NA12878 | Simple Deletions compared to NIST | Svelter | DEL | 1624 | 2314 | 2623 | 999 | 0.7018 | 0.6191 | 0.6579 |
|  |  | Delly | DEL | 1012 | 2314 | 1300 | 288 | 0.4373 | 0.7785 | 0.5600 |
|  |  | Lumpy | DEL | 1706 | 2314 | 2569 | 863 | 0.7373 | 0.6641 | 0.6988 |
|  |  | Pindel | DEL | 1591 | 2314 | 2046 | 455 | 0.6876 | 0.7776 | 0.7298 |
|  |  | erds | DEL | 1409 | 2314 | 1806 | 397 | 0.6089 | 0.7802 | 0.6840 |
|  | Simple Deletions compared to NIST set + Pacbio Validation Set | Svelter | DEL | 2218 | 3672 | 2623 | 405 | 0.6040 | 0.8456 | 0.7047 |
|  |  | Delly | DEL | 1201 | 3672 | 1300 | 99 | 0.3271 | 0.9238 | 0.4831 |
|  |  | Lumpy | DEL | 2268 | 3672 | 2569 | 301 | 0.6176 | 0.8828 | 0.7268 |
|  |  | Pindel | DEL | 1994 | 3672 | 2046 | 52 | 0.5430 | 0.9746 | 0.6974 |
|  |  | erds | DEL | 1735 | 3672 | 1806 | 71 | 0.4725 | 0.9607 | 0.6334 |
| CHM1 | Simple Deletions compared to Chaisson et.al Set | Svelter | DEL | 1188 | 3588 | 1728 | 325 | 0.3311 | 0.6875 | 0.4470 |
|  |  | Delly | DEL | 243 | 3588 | 722 | 479 | 0.0677 | 0.3366 | 0.1128 |
|  |  | Lumpy | DEL | 844 | 3588 | 1524 | 680 | 0.2352 | 0.5538 | 0.3302 |
|  |  | Pindel | DEL | 765 | 3588 | 1106 | 341 | 0.2132 | 0.6917 | 0.3259 |
|  |  | erds | DEL | 447 | 3588 | 1089 | 642 | 0.1246 | 0.4105 | 0.1911 |
|  | Simple Deletions compared to Chaisson et.al + Pacbio Validation Set | Svelter | DEL | 1617 | 3866 | 1728 | 34 | 0.4183 | 0.9358 | 0.5781 |
|  |  | Delly | DEL | 612 | 3866 | 722 | 110 | 0.1583 | 0.8476 | 0.2668 |
|  |  | Lumpy | DEL | 1418 | 3866 | 1524 | 106 | 0.3668 | 0.9304 | 0.5262 |
|  |  | Pindel | DEL | 1068 | 3866 | 1106 | 38 | 0.2763 | 0.9656 | 0.4296 |
|  |  | erds | DEL | 693 | 3866 | 1089 | 396 | 0.1793 | 0.6364 | 0.2797 |

Supplemental Table 3. Running time in hours of different algorithms using post-processed alignments from NA12878. Results were obtained using a single core for each chromosome on a Dell C6100 with 2.67 GHz Intel Xeon X5650 processor with a maximum memory of 8GB.

| chromosome | svelter | delly | lumpy | pindel | erds^#^ |
| --- | --- | --- | --- | --- | --- |
| chr1 | 7.7563 | 1.0311 | 2.0465 | * | 1.8402 |
| chr2 | 7.0589 | 1.0261 | 2.1421 | 12.5220 | 1.8855 |
| chr3 | 5.1461 | 0.8034 | 1.6076 | 6.1863 | 1.5029 |
| chr4 | 4.5744 | 0.8908 | 1.5875 | 8.8362 | 1.4624 |
| chr5 | 4.6423 | 0.7101 | 1.5012 | 5.4523 | 1.3818 |
| chr6 | 4.4831 | 0.6068 | 1.4446 | 6.3549 | 1.3127 |
| chr7 | 3.9558 | 0.5985 | 1.3626 | 6.4219 | 1.2253 |
| chr8 | 3.9148 | 0.6163 | 1.1549 | 4.5544 | 1.1098 |
| chr9 | 3.1073 | 0.4053 | 1.1400 | 6.4134 | 0.9711 |
| chr10 | 3.3541 | 0.9472 | 1.3333 | * | 1.0850 |
| chr11 | 3.4004 | 0.5190 | 1.1100 | 6.5348 | 1.0935 |
| chr12 | 3.8711 | 0.5228 | 1.0619 | 4.5107 | 1.0104 |
| chr13 | 2.1006 | 0.3366 | 0.7194 | 2.9312 | 0.7976 |
| chr14 | 2.1463 | 0.3229 | 0.6918 | 4.5669 | 0.6919 |
| chr15 | 1.9132 | 0.3394 | 0.6742 | 2.5471 | 0.6413 |
| chr16 | 2.4926 | 1.1966 | 0.8031 | 6.8067 | 0.6750 |
| chr17 | 2.7244 | 0.3684 | 0.7396 | 4.9526 | 0.5785 |
| chr18 | 2.1359 | 0.2963 | 0.6178 | 4.8910 | 0.5896 |
| chr19 | 2.2808 | 0.3236 | 0.5499 | 5.4751 | 0.4446 |
| chr20 | 1.9088 | 0.1918 | 0.5021 | 2.6400 | 0.4748 |
| chr21 | 0.9333 | 0.1707 | 0.3271 | 1.9323 | 0.3029 |
| chr22 | 1.0984 | 0.1330 | 0.3045 | 1.4041 | 0.2847 |
| chrX | 0.0177 | 0.5033 | 1.2475 | 5.0937 | 0.9118 |
| chrY | 0.0147 | 0.1002 | 0.0693 | 0.9933 | 0.0000 |
| chrM | 0.0127 | 0.0110 | 0.0753 | 0.3625 | 0.0001 |

*Did not produce results using the allocated resources

^#^Does not include runtime required for mandatory SNP genotyping data


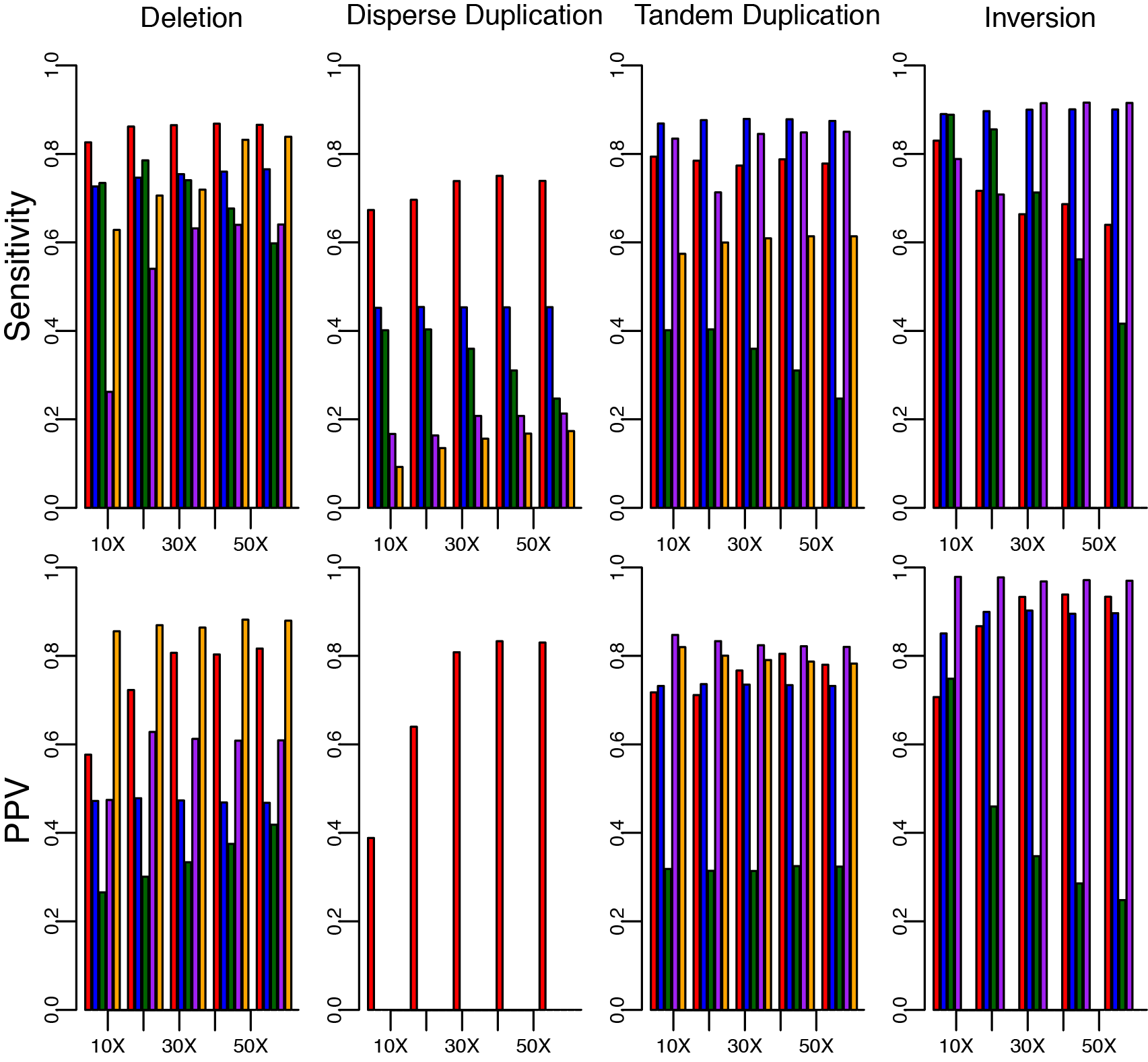
Supplemental Figure 1. Assessment of accuracy on simulated simple events. Sensitivity and positive predictive values for SVelter (red), Delly (blue), Lumpy (green), Pindel (purple), and ERDS (yellow) across different simple SV types and sequence coverage on combined simulated homozygous and heterozygous events. For dispersed duplications, only SVelter was considered for positive predictive values and all predictions by other algorithms that did not overlap simulated results were considered only for the tandem duplication category.


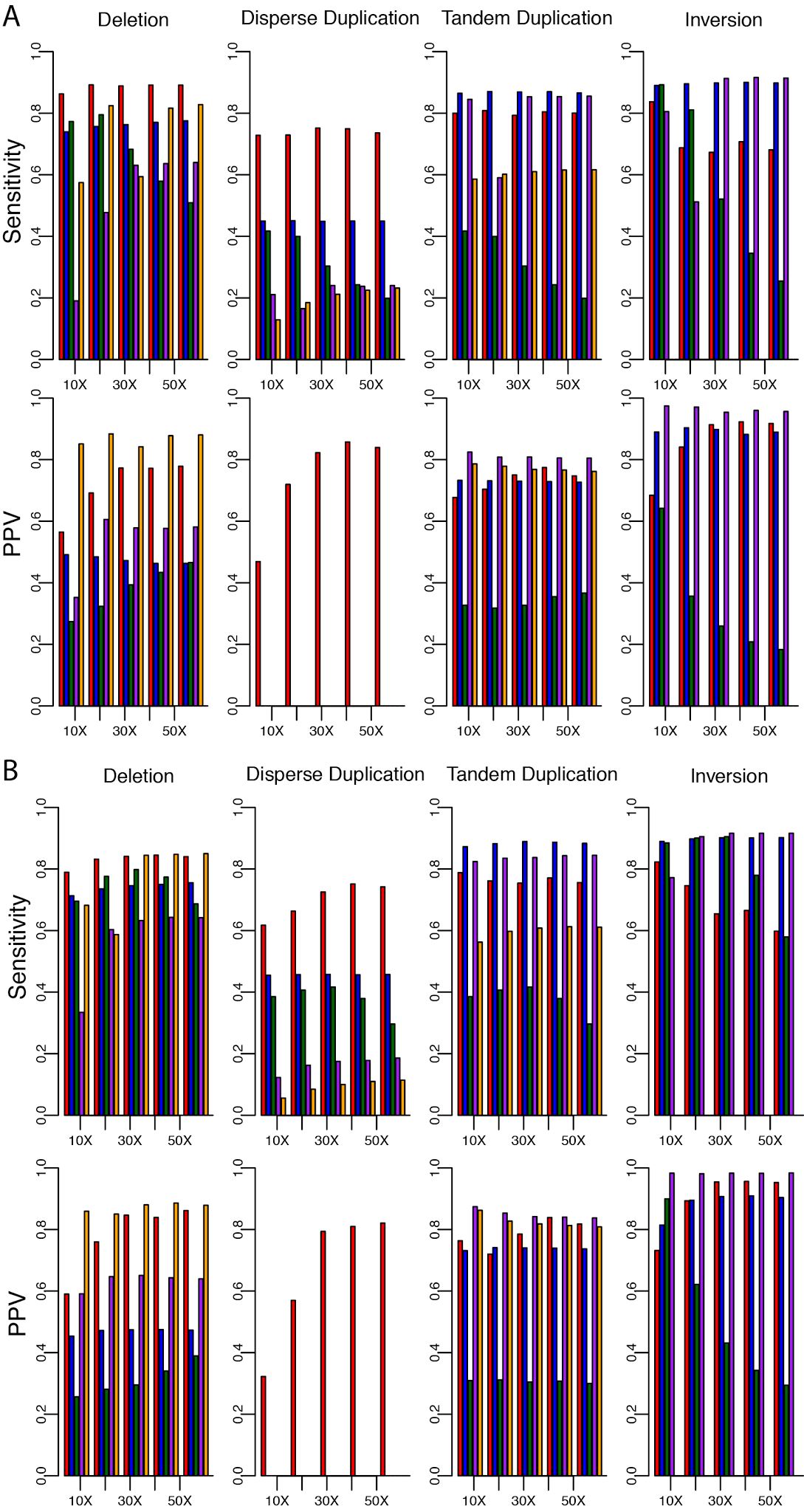


Supplemental Figure 2. Assessment of accuracy on simulated (A) homozygous and (B) heterozygous events. Sensitivity and positive predictive values for SVelter (red), Delly (blue), Lumpy (green), Pindel (purple), and ERDS (yellow) across different simple SV types and sequence coverage. For dispersed duplications, only SVelter was considered for positive predictive values and all predictions by other algorithms that did not overlap simulated results were considered only for the tandem duplication category.


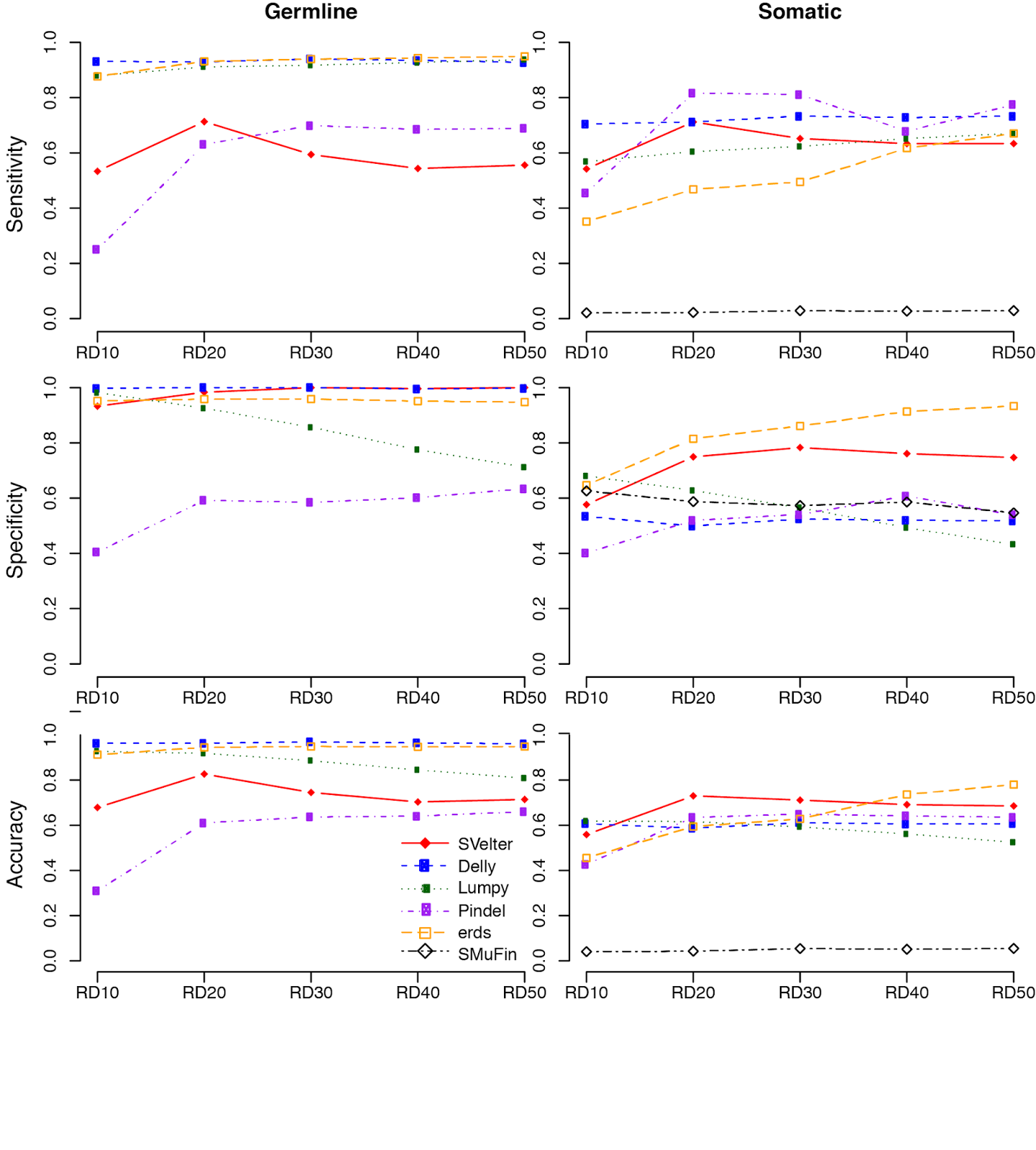


Supplemental Figure 3. Assessment of accuracy on simulated tumor and matched normal genomes. Sensitivity, specificity and combined accuracy (F-Score) of multiple algorithms at different coverage using simulated matched germline and somatic data generated locally using the Moncunill et al, 2014 set of variant calls and simulation strategy. SMuFin is absent in germline data as it only reports somatic events and is only reported for chromosomes 16-22.


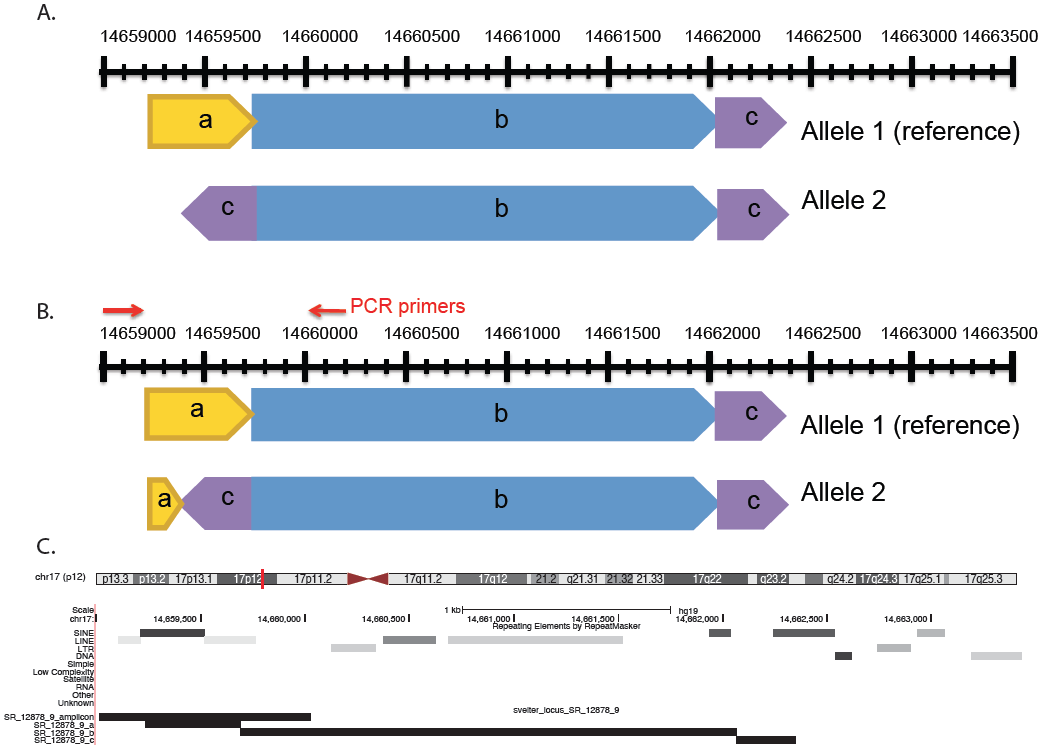


Supplementa Figure 4. Validation of inverted duplication (chr17_14659237_14662064_14662349_14662516_C) on at locus chr17:14659000-14663500. (A) Predicted structure of CSV. (B) PCR primer strategy and resulting observed structured. Primer sequences S91: GTGCACAGGATTGCTTCTGA, S92: TGTGTGGCTTTGACCACAAT. (C) Graphical representation of predicted structure and observed PCR product.


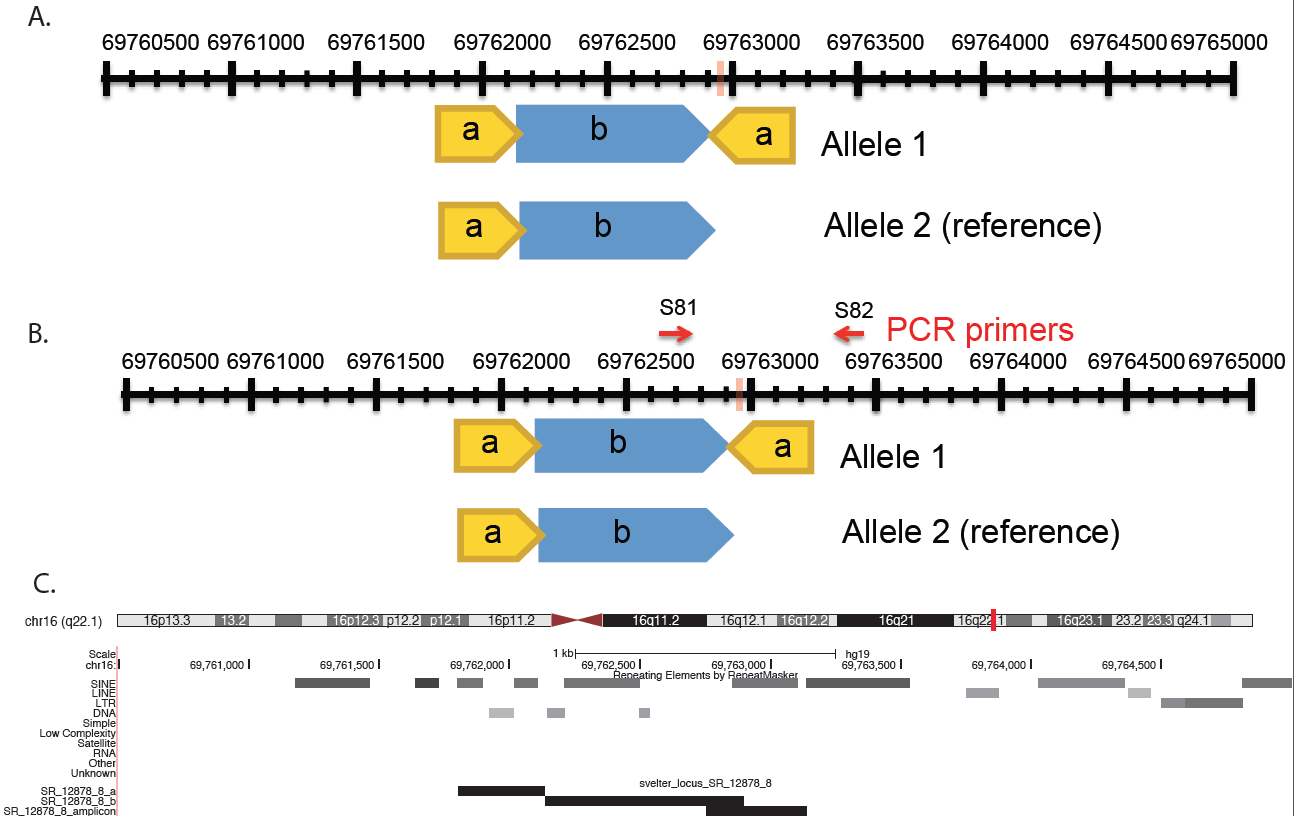


Supplemental Figure 5. Validation of inverted duplication (chr16_69761804_69762136_69762896_69766900_C) on at locus chr16:69760500-69765500. (A) Predicted structure of CSV. (B) PCR primer strategy and resulting observed structured. Primer sequences S81: CCCATCCCAAGTCATCTCAT, S82: AAATGTCTGTCTTTACCACTGTGTAG. (C) Graphical representation of predicted structure and observed PCR product.


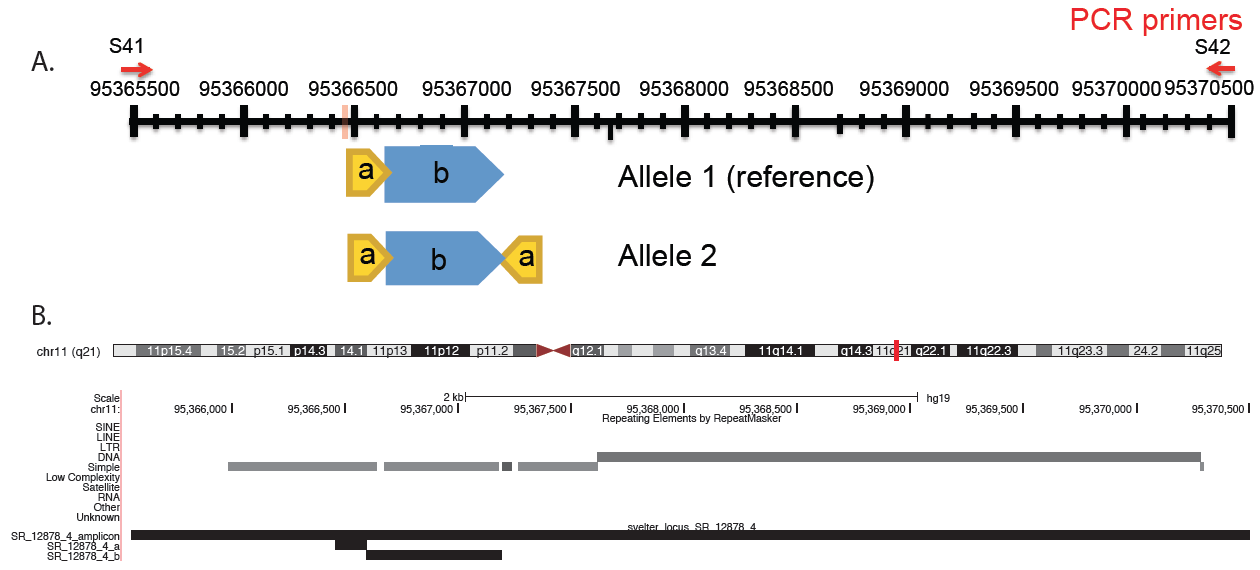


Supplemental Figure 6. Attempted validation of inverted duplication (chr11_95366462_95366593_95367193_C) at locus chr11:95365500-95375000. This region contained numerous repetitive elements making direct PCR validation difficult such that no distinct bands were produced resulting in an inconclusive validation status. (A) PCR primer strategy and predicted structured. Primer sequences S41: GCCAGGCAGTCAGAATTAGC, S42: TCCCTGAGGACAGGAACAAC. (B) Graphical representation of predicted structures.


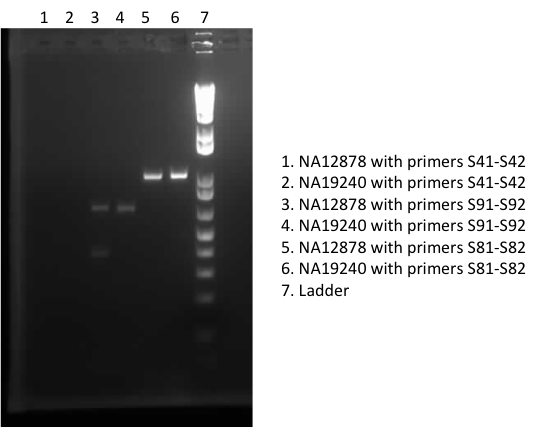


Supplemental Figure 7. Electrophoresis GEL image of PCR products for each primer pair in two different samples (NA12878, NA19240), as outlined in Supplemental Figures 1-3.


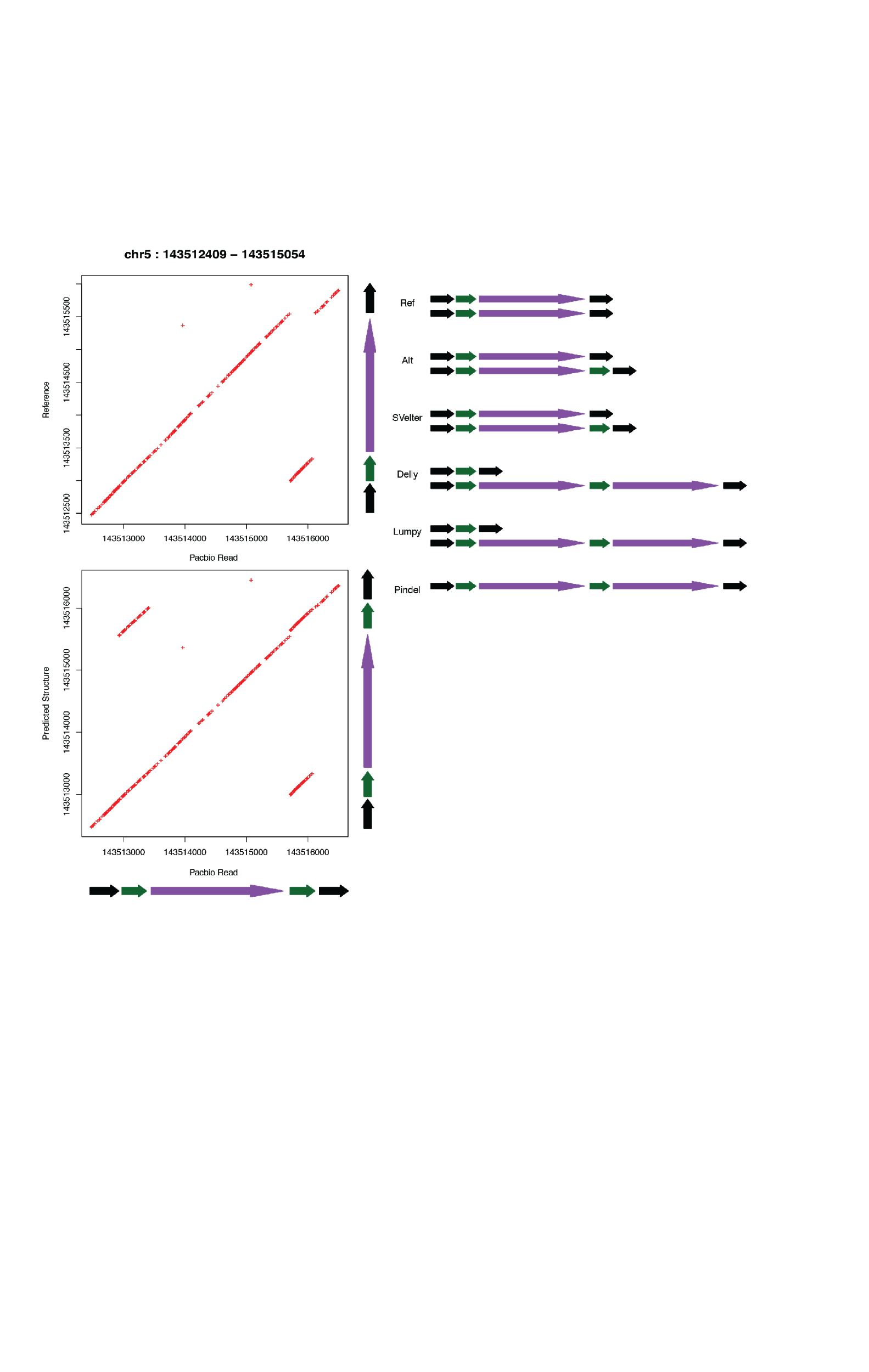


Supplemental Figure 8. Dot plot of a PacBio read (fa716c69_55756_0) from NA12878 against both unaltered reference sequence (chr5:143512409-143515054) and modified reference sequence containing the predicted rearrangement. Colored arrows on the right side indicate reference and alternative structures as diploid arrangements, as well as predictions from each individual algorithm. Ploidy for individual approaches is based on reported genotypes where available.


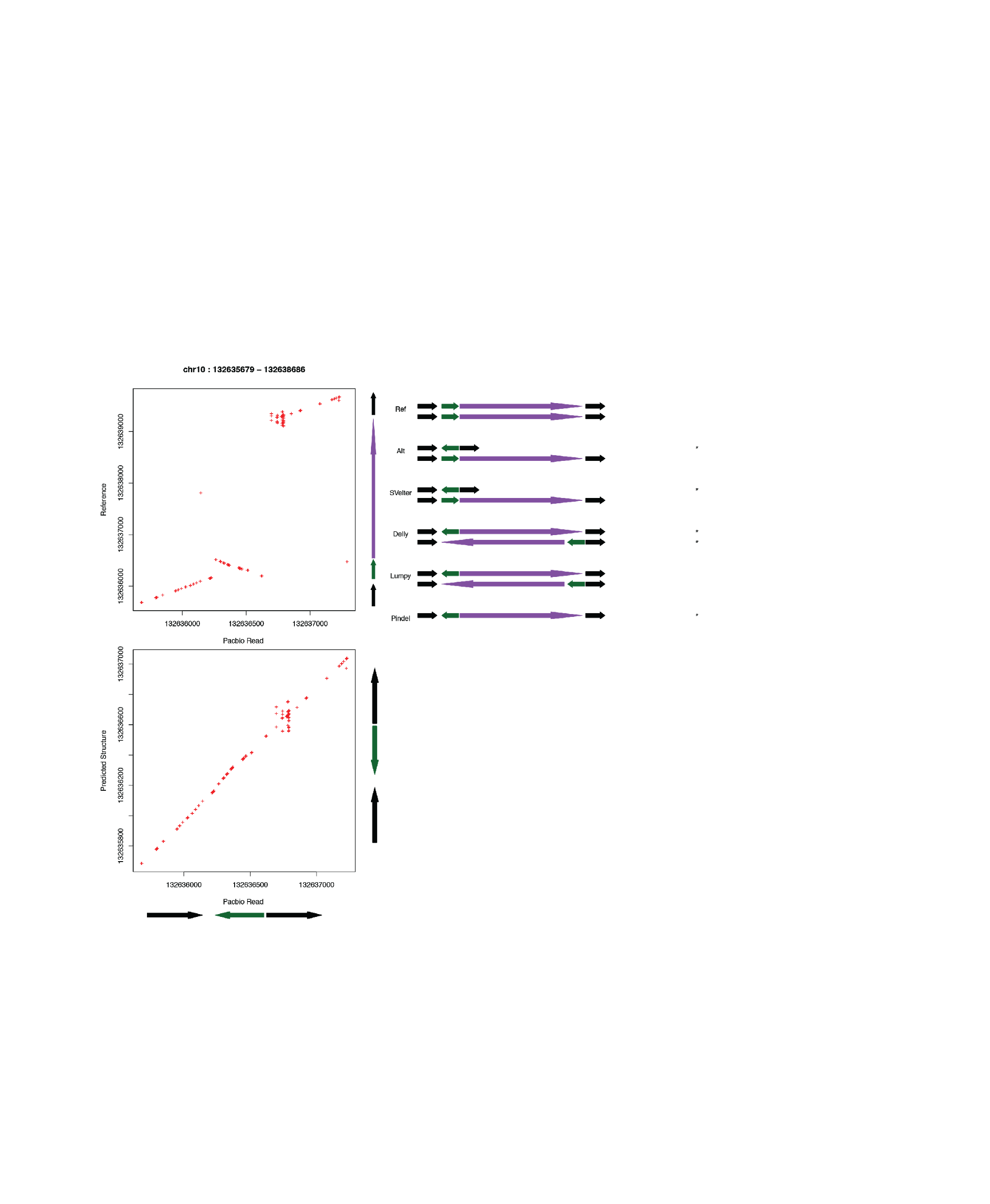


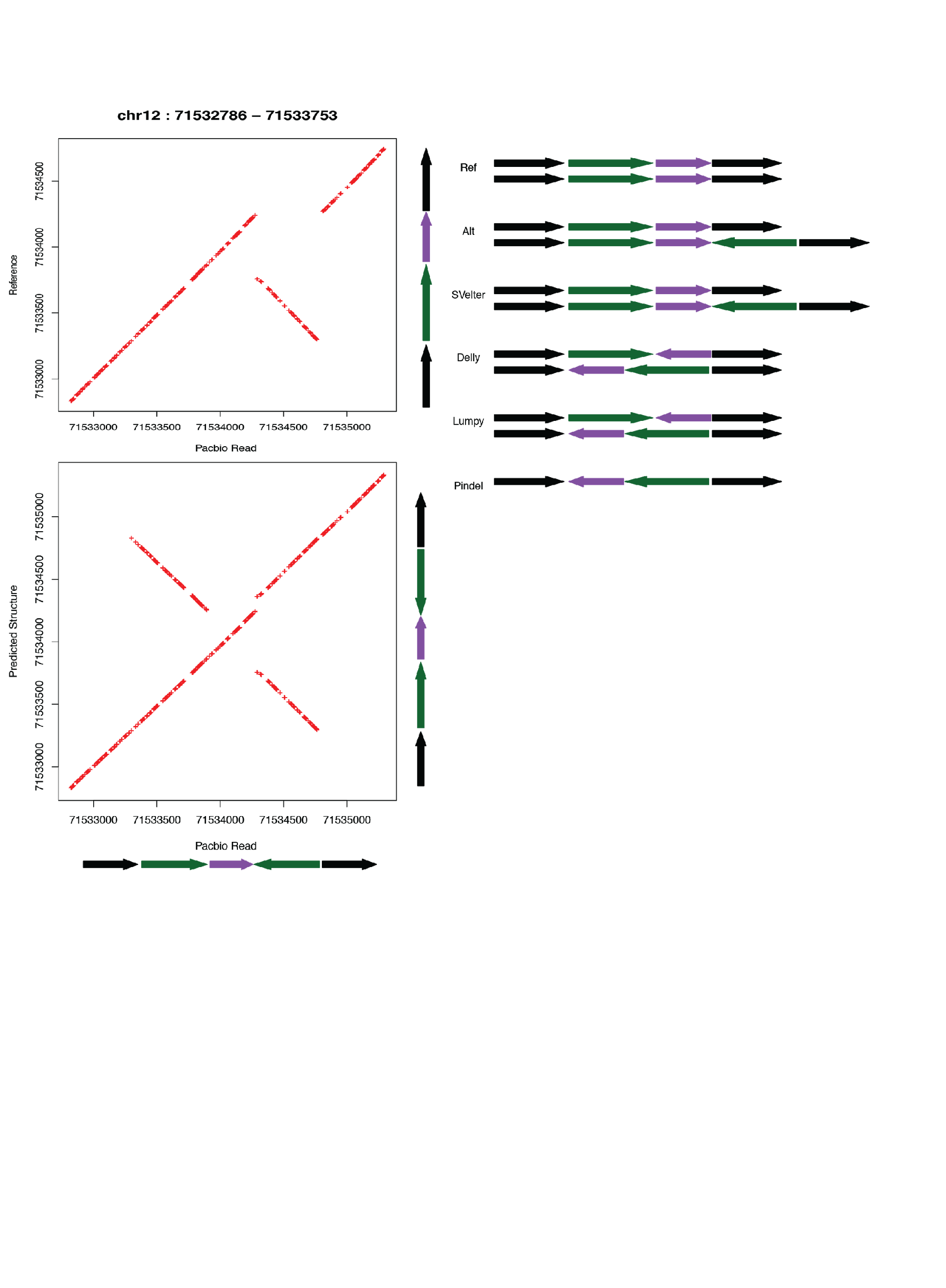


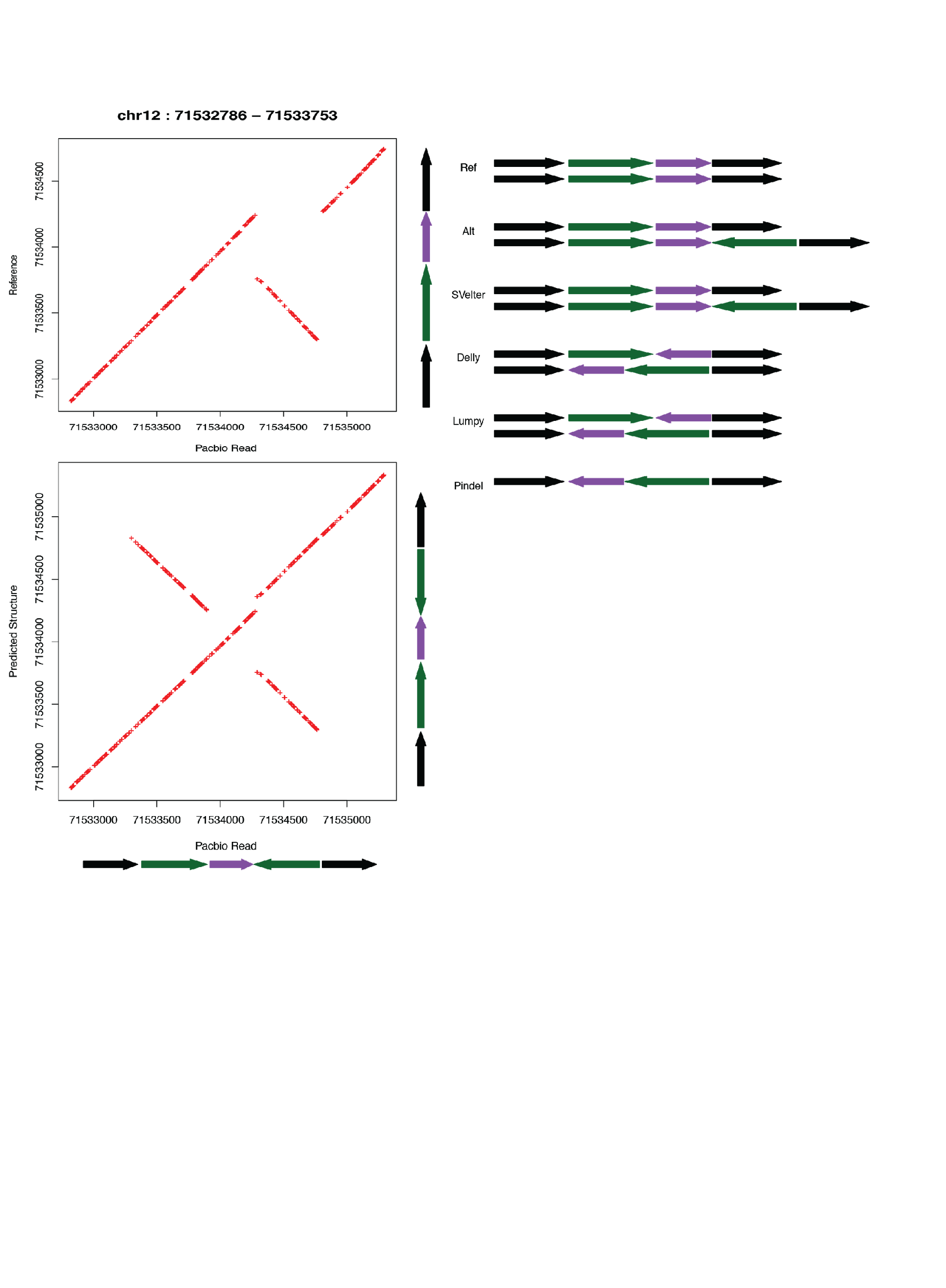


Supplemental Figure 9. Dot plot of a PacBio read (5308fbec_46356_10341) from NA12878 against both unaltered reference sequence (chr12:71532786-71533753) and modified reference sequence containing the predicted rearrangement. Colored arrows on the right side indicate reference and alternative structures as diploid arrangements, as well as predictions from each individual algorithm. Ploidy for individual approaches is based on reported genotypes where available.


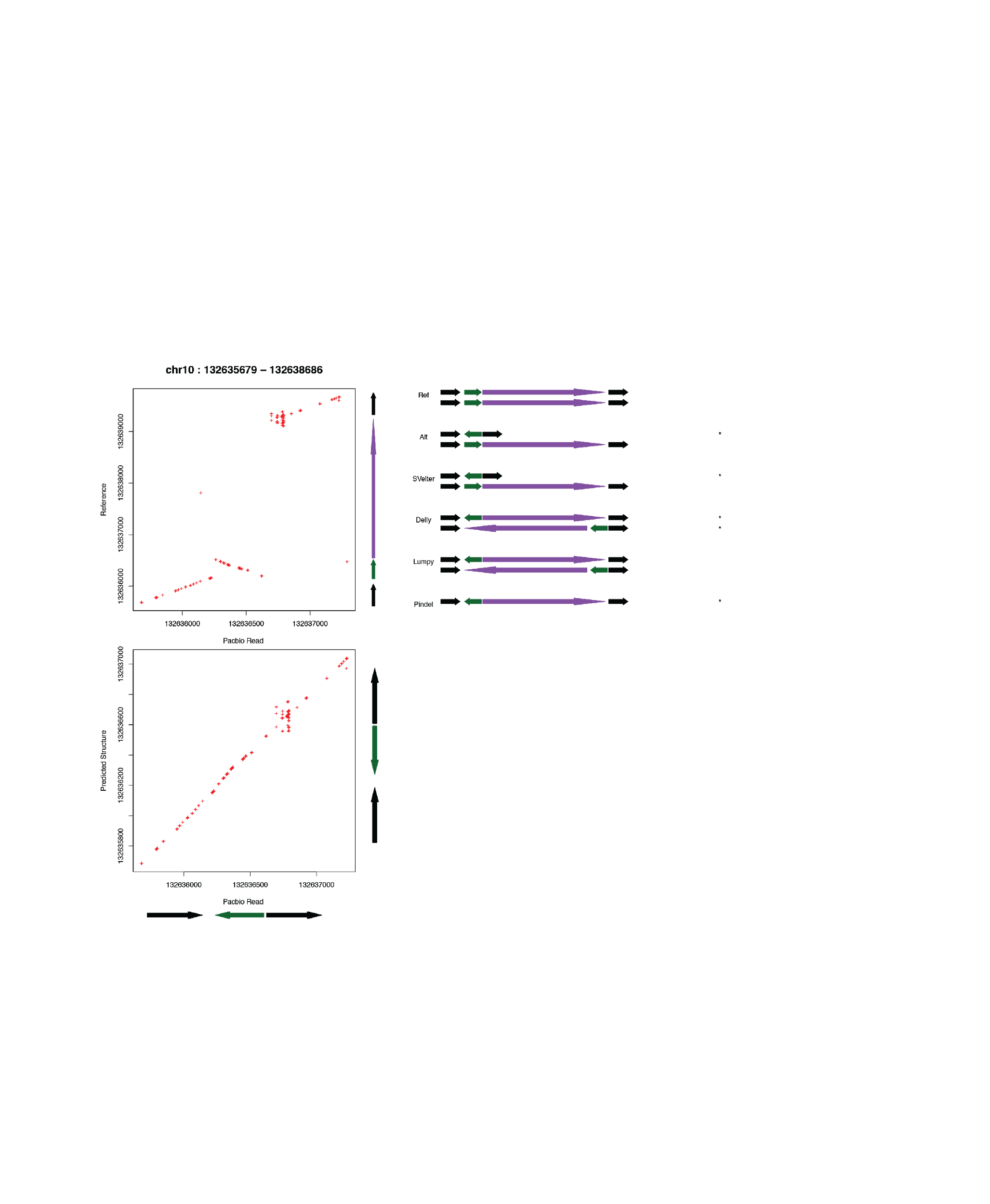


Supplemental Figure 10. Dot plot of a PacBio read (325320e3_146839_203) from NA12878 against both unaltered reference sequence (chr10:132635679-132638686) and modified reference sequence containing the predicted rearrangement. Colored arrows on the right side indicate reference and alternative structures as diploid arrangements, as well as predictions from each individual algorithm. Ploidy for individual approaches is based on reported genotypes where available.


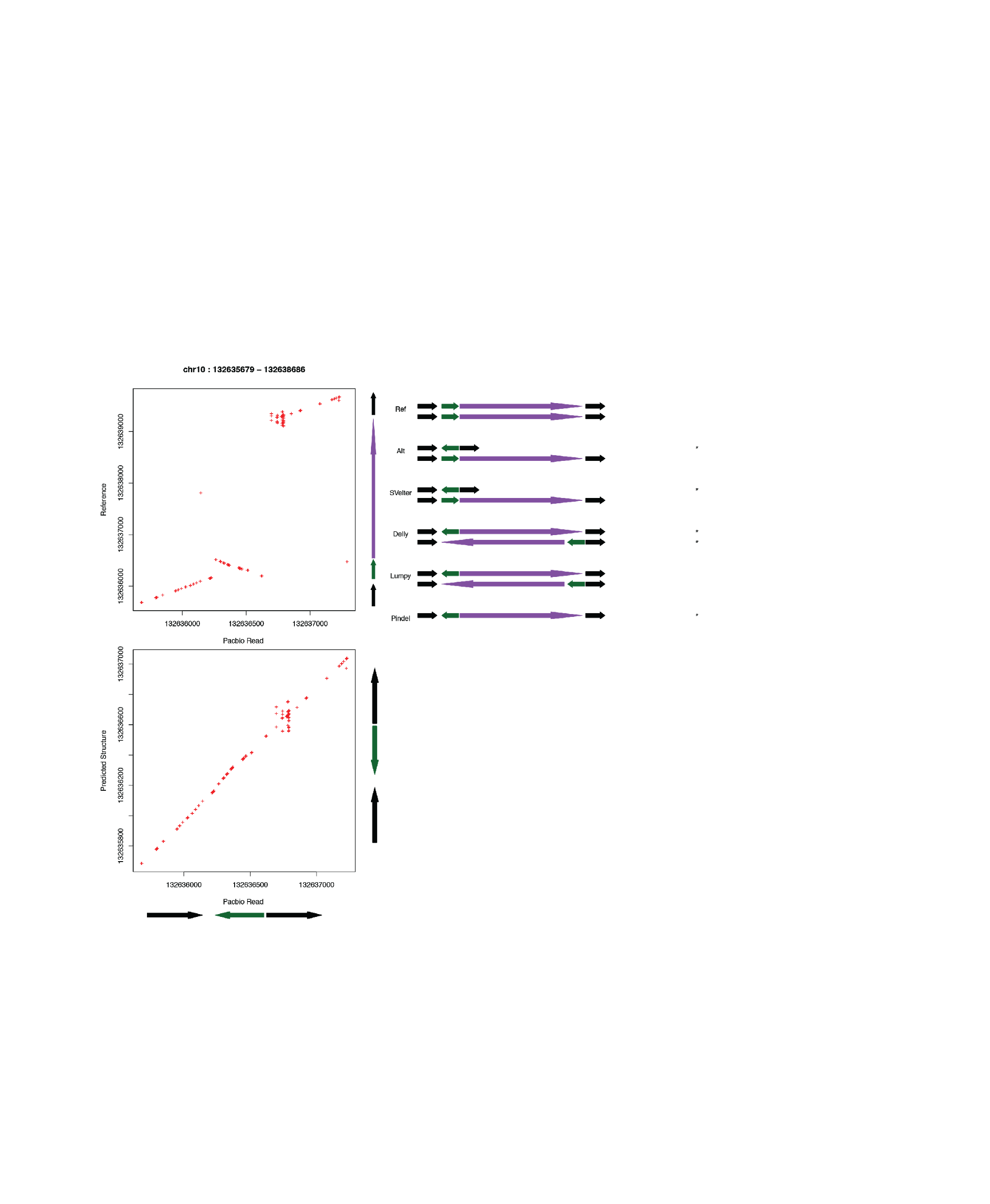


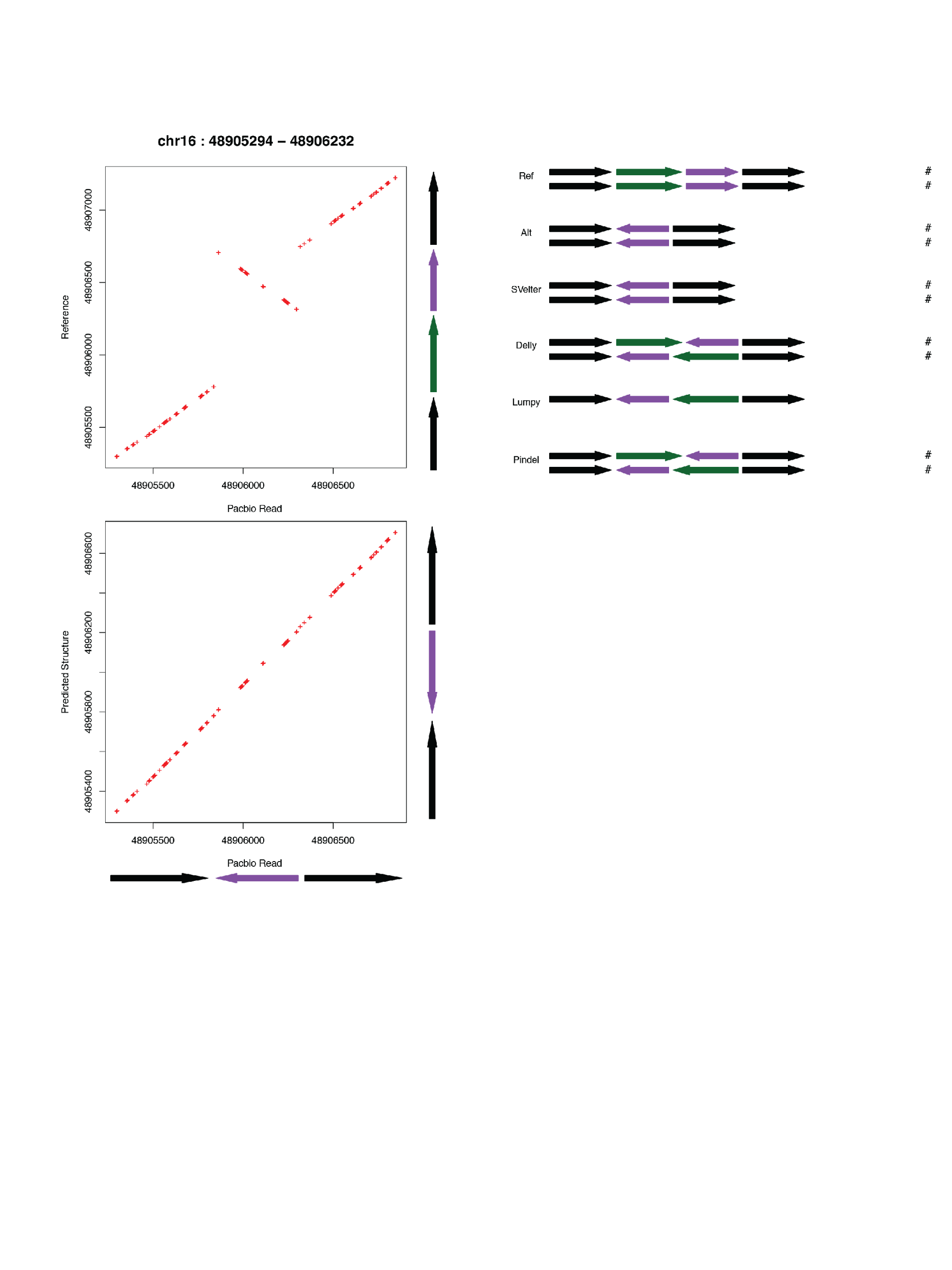


Supplemental Figure 11. Dot plot of a PacBio read (fffb5d0d_36049_18160) from NA12878 against both unaltered reference sequence (chr16:48905294-48906232) and modified reference sequence containing the predicted rearrangement. Colored arrows on the right side indicate reference and alternative structures as diploid arrangements, as well as predictions from each individual algorithm. Ploidy for individual approaches is based on reported genotypes where available.
