# Supplementary material for: Resolving complex structural genomic rearrangements using a randomized approach
Source: Genome Biol. 2016 Jun 10;17:126. doi: 10.1186/s13059-016-0993-1 (PMC4901421; doi:10.1186/s13059-016-0993-1)
Supplement: Additional file 3: — Supplemental Methods outlining the software and parameter usage that was used to generate the presented results. (DOCX 10 kb) [file 13059_2016_993_MOESM3_ESM.docx]

**To produce and process simulate data for evaluation:**

(Additional documentation available at: <https://github.com/mills-lab/svelter>)

**Step1.**

**We used our own script: *Produce.Simulated.FussyJuncs.py* to modify the germline reference genome with pre-designed simple and complex SVs in both homozygous and heterozygous status. Output files includes altered reference genomes in fasta format, and detailed record of implemented SVs.**

**We also adopted the simulated cancer and matched normal references reported by *Moncunill et al* to evaluate performance of SVelter on somatic events. Those references are publicly available from http://cg.bsc.es/smufin/.**

***Produce.Simulated.FussyJuncs.py***

**Usage:**

Produce.Simulated.FussyJuncs.py [options] <parameters>

**Options:**

heterozygous: simulate simple heterozygous SVs

homozygous: simulate simple homozygous SVs

complex: simulate complex SVs

**Parameters:**

--reference: reference genme

--input-sim: input sim format,see example

--input-rec: input rec format, specially designed for complex events,see example

--output-prefix: prefix of output files

**Examples:**

Produce.Simulated.FussyJuncs.py heterozygous --reference genome.fa --input-sim het.sim --output-prefix ..output/path/simple_het

Produce.Simulated.FussyJuncs.py homozygous --reference genome.fa --input-sim homo.sim --output-prefix ..output/path/simple_homo

Produce.Simulated.FussyJuncs.py complex --reference genome.fa --input-sim comp_het.sim --input-rec comp_het.rec --output-prefix ..output/path/comp_het

Produce.Simulated.FussyJuncs.py complex --reference genome.fa --input-sim comp_homo.sim --input-rec comp_homo.rec --output-prefix ..output/path/comp_homo

*Example of .sim and .rec file could be found under:*

*https://github.com/mills-lab/svelter/tree/master/Support.Materials/ExampleFiles*

**Step2.**

**We used *wgsim* to simulate paired-end sequences up to different read depth**

**Here are the parameters we used in the manuscript:**

wgsim -e 0.001 -d 500 -s 100 -N num.of.reads -1 101 -2 101 -r 0.001 -R 0.1 -X 0 altered.reference.fa out1.fq out2.fq

**Besides, we also used *art_illumina* to simulate paired-end sequences from simulated somatic and matched germline references up to different read depth.**

**We keep the simulation consist with what was reported by *Moncunill et al*.**

art_illumina -sam -i altered.reference.fa -p -l 80 -ss HS20 -f 20 -m 500 -s 20 -o output

**Step3.**

**We used bwa mem to align simulated reads to reference genome, and then sort and index the output files with samtools.**

bwa mem genome.fa input.1.fq input.2.fq > input.sam

samtools view -h -Sb input.sam -o input.bam

samtools sort input.bam input.sorted

samtools index input.sorted.bam

**Step4.**

**Four algorithms were applied on simulated data with following commands:**

**SVelter**

SVelter.py Index --exclude Exclude.genome.bed --reference genome.fa --workdir workdir/directory --copyneutral CN2.genome.bed --svelter-path ../svelter --ref-index ../pre-indexed/ref/if/provided/by/SVelter

SVelter.py --workdir workding/directory --sample input.bam

**Delly**

delly -t [SV type] -x human.hg19.excl.tsv -o Delly.input.vcf -g genome.fa input.sorted.bam

**Lumpy**

samtools view input.sorted.bam| ../lumpy-sv/scripts/split_unmapped_to_fasta.pl -b 20 > Lumpy.input.um.fq

bwa bwasw -H -t 20 genome.fa Lumpy.input.um.fq | samtools view -Sb -> Lumpy.input.sr.bam

samtools sort Lumpy.input.sr.bam Lumpy.input.sr.sorted

samtools index Lumpy.input.sr.sorted.bam

samtools view input.bam | tail -n+100000 | ../lumpy-sv/scripts/pairend_distro.py -r 101 -X 4 -N 10000 -o Lumpy.input.histo

../lumpy-sv/bin/lumpy -mw 4 -tt 0.0 -x Exclude.bed -pe bam_file:input.sorted.bam,histo_file:/Lumpy.input.histo ,mean:ILMean ,stdev:ILStd,read_length:101,min_non_overlap:101,discordant_z:4,back_distance:20,weight:1,id:bwa,min_mapping_threshold:20 –sr bam_file:Lumpy.input.sr.sorted.bam,back_distance:20,weight:1,id:bwa,min_mapping_threshold:20 > Lumpy.input.pesr.bedpe

**Pindel**

pindel -f genome.fa -i input.config.txt -c ALL -o input.bam.pindel

pindel2vcf -P input.bam.pindel -r genome.fa -R genome -d date -v Pindel.input.vcf

**Erds**

java -jar GenomeAnalysisTK.jar -T UnifiedGenotyper -R genome.fa -I input.bam -o input.geli.calls.vcf

perl ../erds/erds_pipeline.pl -b input.bin -v input.geli.calls.vcf -r genome.fa -o output/path/

**We also applied SMuFin on the tumor and matched normal datasets with these commands:**

**SMuFin:**

mpirun --np 30 SMuFin --ref hg19.fa --normal_fastq_1 SMuFin_Normal_1.txt --normal_fastq_2 SMuFin_Normal_2.txt --tumor_fastq_1 SMuFin_Tumor_1.txt --tumor_fastq_2 SMuFin_Tumor_2.txt --patient_id patient_id --cpus_per_node 30

**Step5a. Evaluate different SV calling algortihms on Simulated Simple events. We used our own script: *SV.Simple.Output.Process.py* and some shell commands in pre-processing step:**

**Usage:**

SV.Simple.Output.Process.py [options] <parameters>

**Options:**

vcf-to-bed: extract simple SVs from vcf files and output in separate bed files

bedpe-to-bed: extract simple SVs from bedpe files and output in separate bed files

Mappable-Control: remove SVs located outside mappable regions

Size-Control: filter out SVs of size outside defined range

TRA-Control: remove SVs overlap with defined SVs

**Parameters for vcf-to-bed:**

--input: input file

**Parameters for bedpe-to-bed**

--input: input file

--reference: reference.genome.fa

**Parameters for Mappable-Control:**

--input: input file

--ref-prefix: reference.genome.fa

**Parameters for Size-Control:**

--input: input.bed

--min-size: reference.Mappable. describing mappable regions

--max-size: reference.Mappable. describing mappable regions

**Parameters for TRA-Control:**

--input: input.bed

--TRA-rec: TRA information kept in .rec files

**Examples:**

grep -v LowQual Input.vcf>Input_QC.vcf-to-bed

#Remove calls that failed quality contrl

SV.Simple.Output.Process.py vcf-to-bed --input Input_QC.vcf

#Extract simple SVs from vcf format and put them in bed ffiles

SV.Simple.Output.Process.py bedpe-to-bed --input Input_QC.bedpe --reference genome.fa #Extract simple SVs from bedpe format and put them in bed ffiles

SV.Simple.Output.Process.py Mappable-Control --input Input_QC.DEL.bed --ref-prefix genome.Mappable.bed

#keep calls that fall within mappable genomic regions that defined by SVelter

SV.Simple.Output.Process.py TRA-Control --TRA-rec ref.TRA.rec --input Input_QC.DEL.Mappable.bed #remove calls overlap with implemented TRAs

SV.Simple.Output.Process.py Size-Control --min-size 100 --max-size 1000000000 --input Input_QC.DEL.Mappable.TRAFree.bed #Keep SV calls within certain size range

Produce.Pseudo.ROC.stats.py --path_ref /path/where/predicted/SVs/located/ --path_in /path/to/reference/bed/files/ --appdix .Mappable.TRAFree.min100.max1000000000.bed

Produce.Barplot.For.Simple.Simu.R

Pseudo.ROC.Mappable.TRAFree.min100.max1000000000.Stats

#Input.Stats were produced by previous step

**Step5b. Evaluate different SV calling algortihms on Simulated Complex events. We used our own script: SV.Complex.Output.Process.py:**

**Usage:**

SV.Complex.Output.Process.py [options] <parameters>

**Options:**

SVelter

Delly

Lumpy

Pindel

report2stat

comparison

**Parameters for SVelter/Delly/Lumpy/Pindel:**

--reference: reference genome

--input-path: path where outputs were kept

--ref-sv: pre set complex SVs to compare to

**Parameters for report2stat:**

--reference: reference genome

--report: .report files

**Parameters for comparison:**

--path: folder contains all .report files

**Parameters for stat-integrate:**

--stat: .stat file

**Examples:**

SV.Complex.Output.Process.py SVelter --reference genome.fa --input-path /path/to/SVelter/output --ref-sv comp_het.SV.rec

SV.Complex.Output.Process.py Delly --reference genome.fa --input-path /path/to/Delly/output --ref-sv comp_het.SV.rec

SV.Complex.Output.Process.py Lumpy --reference genome.fa --input-path /path/to/Lumpy/output --ref-sv comp_het.SV.rec

SV.Complex.Output.Process.py Pindel --reference genome.fa --input-path /path/to/Pindel/output --ref-sv comp_het.SV.rec

SV.Complex.Output.Process.py erds --reference genome.fa --input-path /path/to/Pindel/output --ref-sv comp_het.SV.rec

SV.Complex.Output.Process.py report2stat --reference genome.fa --report input.report

SV.Complex.Output.Process.py comparison --path /folder/contains/all/report/files

**To process real data (NA12878/CHM1) for evaluation:**

**Step1. We apply different algorithms on NA12878 with following settings:**

**SVelter**

SVelter.py Setup --workdir ./ --reference hg19.fa --exclude ../svelter/Support/

Exclude.hg19.bed --copyneutral ../svelter/Support/CN2.hg19.bed --svelter-path ../svelter/ --ref-index ../svelter/Index.Reference/hg19/ --prefix SVelter.NA12878

**Delly**

delly -t SV -s 10 -x human.hg19.excl.tsv -o Delly.SV.NA12878_S1.test.DEL.vcf -g hg19.fa NA12878.bam

**Lumpy**

samtools view NA12878.bam ../lumpy-sv/scripts/split_unmapped_to_fasta.pl -b 20 > NA12878.um.fq

bwa bwasw -H -t 20 hg19.fa NA12878.um.fq | samtools view -Sb -> NA12878.sr.bam

samtools sort NA12878.sr.bam NA12878.sr.sorted

samtools index NA12878.sr.sorted.bam

samtools view NA12878.bam | tail -n+100000 | ../lumpy-sv/scripts/pairend_distro.py -r 101 -X 4 -N 10000 -o Lumpy1.NA12878.histo

../lumpy-sv/bin/lumpy -mw 4 -tt 0.0 –x Exclude.bed -pe bam_file: NA12878.bam, histo_file: Lumpy1.NA12878.histo, mean:290.505405405, stdev:110.366312087, read_length:101, min_non_overlap:101, discordant_z:4, back_distance:20, weight:1, id:bwa, min_mapping_threshold:20 –sr bam_file: NA12878.sr.sorted.bam, back_distance:20, weight:1, id:bwa, min_mapping_threshold:20 > Lumpy.NA12878.pesr.bedpe

**Pindel**

pindel -f hg19.fa -i NA12878.config.txt -c ALL -o NA12878.pindel

pindel2vcf -P NA12878.pindel -r hg19.fa -R hg19 -d 2015 -v Pindel.NA12878.vcf

**Step2. We compare simple deletions reported by each algorithm against the GIAB set with the same approach described above**

**Step3. We used our own python script:** ***Pacbio.Vali.py* to validate all simple deletions reported by each algorithm**

**Usage:**

Pacbio.Validation.py [options] <parameters>

**Options:**

Simple, for simple events [DEL,DUP,INV] validation

Complex, for complex events validation

svelter-to-rec, to transfer .svelter to .vali format

**Parameters for simple / complex:**

--bam-file, input pacbio data in bam format

--reference, reference genome in fasta format

--vali-file, files recording predictions in .vali format

--path-delly, folder where delly output were stored, only for complex

--path-lumpy, folder where lumpy output were stored, only for complex

--path-pindel, folder where pindel output were stored, only for complex

--output-path, folders where output files, including necessary interval files, will be written to

--window-size, window size for read comparison, defalut 10

**Parameters for svelter-to-rec**

--input, input file in .svleter format

--output, output file name in .vali format. if not specified, would be named the same with input with differnet appdix

--qc-structure, minimum quality score of a resolved structure to be considered as PASS and included in the output vcf file, default -20

*Example of.vali file could be found under:*

[*https://github.com/mills-lab/svelter/tree/master/Support.Materials/ExampleFiles*](https://github.com/mills-lab/svelter/tree/master/Support.Materials/ExampleFiles)

**Examples:**

Pacbio.Validation.py svelter-to-rec --input SVelter.output.svelter --output SVelter.output.vali --qc-structure -20

Pacbio.Validation.py simple –bam-file pacbio.bam --reference genome.fa --vali-file SVelter.output.vali --output-path output/folder/ --window-size 10

Pacbio.Validation.py complex –bam-file pacbio.bam --reference genome.fa --vali-file SVelter.output.vali --path-delly delly/output/folder/ --path-lumpy --path-pindel --output-path output/folder/ --window-size 10
